# Twist1/Dnmt3a and miR186 establish a regulatory circuit that controls inflammation-associated prostate cancer progression

**DOI:** 10.1038/oncsis.2017.16

**Published:** 2017-04-10

**Authors:** X Zhao, R Deng, Y Wang, H Zhang, J Dou, L Li, Y Du, R Chen, J Cheng, J Yu

**Affiliations:** 1 Shanghai Key Laboratory of Tumor Microenvironment and Inflammation, Department of Biochemistry and Molecular Cell Biology, Shanghai Jiao Tong University School of Medicine, Shanghai, China; 2 State Key Laboratory of Oncogenes and Related Genes, Shanghai Jiao Tong University School of Medicine, Shanghai, China; 3 Department of Pathophysiology, Key Laboratory of Cell Differentiation and Apoptosis of Chinese Ministry of Education, Shanghai, China

## Abstract

Increasing evidences suggest that inflammatory microenvironment has a crucial role in prostate cancer (PCa) progression; however, the underlying mechanisms are unclear. Here, we used the inflammation-associated prostate cellular transformation model to screen out a crucial microRNA, miR186, which was significantly downregulated in the transformed cells and effectively rescued the transformed phenotype. On stimulation of inflammatory cytokines, the activated nuclear factor kappa B (NF-κB)/p65 was able to induce miR186 expression through binding to its promoter in non-transformed cells, whereas this pathway was lost in transformed cells. Interestingly, Twist1, which is a reported downstream target of miR186, was responsible for the loss of NF-κB/p65-miR186 pathway. Twist1 downregulated miR186 expression in a novel negative feedback loop binding to the E-box and simultaneously recruiting Dnmt3a, which facilitated the site-specific CpG methylation of the miR186 promoter, thereby blocked the transcriptional activity of NF-κB/p65 and the responsiveness of miR186 to inflammatory signals. The high level of Twist1 triggered this feedback loop that underlies the epigenetic switch, which was essential for maintaining transformed and advanced PCa state. Finally, our clinical data confirmed that the CpG methylation and miR186 expression levels were closely related with inflammation-associated human PCa progression.

## Introduction

Prostate cancer (PCa) has become the most frequently diagnosed cancer and the second leading cause of cancer-related deaths in men in western countries.^1^ The causes of PCa have not yet been clarified. Prostate carcinogenesis is involved in a series of genetic, epigenetic and environmental alterations, especially inflammatory microenvironment changes. Inflammation has long been associated with the development of cancer,^2, 3^ and approximately 20% of all adult human cancers in organs, such as stomach and liver, result from chronic inflammation.^4^ There are emerging evidences that chronic infection and inflammation can initialize PCa and promote its development.

The nuclear factor kappa B (NF-κB) and microRNAs (miRNAs) pathways have emerged as having crucial roles in inflammation, infection and cancer development.^2, 5, 6, 7, 8, 9, 10^ NF-κB as a transcription factor, which can regulate the expressions of many oncogenes and activate different pro-inflammatory cytokines, chemokines and miRNAs, is a key molecular link between inflammation and tumor initiation and progression.^11^ Twist1 is a known cytokine-responsive target of NF-κB,^12^ and notably NF-κB alone is sufficient for the transcriptional activation of Twist1 in cancer.^13^ On the other hand, the expression of miRNAs is strictly regulated and they can function as immunomodulators. Dysregulation of miRNAs in cancer has been shown to be associated with epigenetic alterations or transcriptional/post-transcriptional mechanisms.^14^ Moreover, the expressions of several miRNAs can be regulated by inflammatory stimulus.^15^ For example, miR155 can be induced by NF-κB in macrophages,^16, 17^ whereas miR21 is induced by Stat3, a transcription factor activated by IL-6.^18^ However, it is not well defined how the cross talk between NF-κB and miRNA can modulate all stages from chronic/nonresolving inflammation to initiation and progression of PCa.

Promoter methylation is tightly associated with gene transcriptional repression, because it may affect the binding affinity of transcription factors (TFs) such as CTCF^19^ and Sp1.^20^ Methylation of CpG at the promoter region can be catalyzed by DNA methyltransferases (Dnmts) including Dnmt1 and Dnmt3a/3b. Dnmt1 functions in maintenance of the established DNA methylation signature, whereas Dnmt3a/3b *de novo* methylate the cytosine residue of CpG in a background of unmethylated DNA.^21^ The mechanisms for targeting specific CpG islands for methylation by Dnmts are incompletely understood but at least clarified that it depends on interactions between some key interacting factors. As Dnmts themselves have no substrate specificity, it is unclear how they are recruited to the proximal region of the gene promoter, thereby to mediate the site-specific CpG methylation for transcriptional repression.

In this study, we established a chronic inflammation-associated PCa model of benign prostatic hyperplasia (BPH) epithelial cell line BPH1/LT-BPH1 (LPS long-term treated BPH1) and combined with another cellular transformation model of P69/M12,^22, 23^ to screen out a crucial miRNA, miR186, which was significantly downregulated in the malignant transformed cells LT-BPH1 and M12 rather than in their parental cells BPH1 and P69, respectively, and its ectopic expression could rescue the transformed phenotypes. In particular, we demonstrated that NF-κB/p65 activation on stimulation of inflammatory cytokines induced the miR186 expression through direct binding to its promoter in the non-transformed BPH1, but not in the chronic inflammation-transformed LT-BPH1. Twist1, which is highly expressed in 90% of PCa tissues and positively associated with PCa Gleason grading,^24^ is a key target of miR186 in PCa^25^ and ovarian cancer.^26^ We have previously demonstrated that miR186 greatly suppresses tumor formation and metastasis *in vitro* and *in vivo* by downregulation of its target Twist1, and the miR186 expression level is significantly decreased and negatively correlated with Twist1 in clinical PCa specimens.^25^ In this study, we further showed that Twist1 repressed miR186 expression by a novel negative feedback loop, in which Twist1 could recruit Dnmt3a to the miR186 promoter to facilitate the site-specific CpG methylation, subsequently blocking the transcriptional activity of NF-κB/p65. During PCa progression, Twist1 upregulation and miR186 downregulation become a stable event with the feedback of miR186/Twist1 maintaining the cell malignantly transformed phenotype and promoting metastasis. Our findings not only contribute to further understand the link between inflammatory environment and PCa progression, but also have important implications for the diagnosis, prognosis and treatment of PCa.

## Results

### MiR186 is a key factor in inflammation-associated transformation of PCa

**Figure 1:**
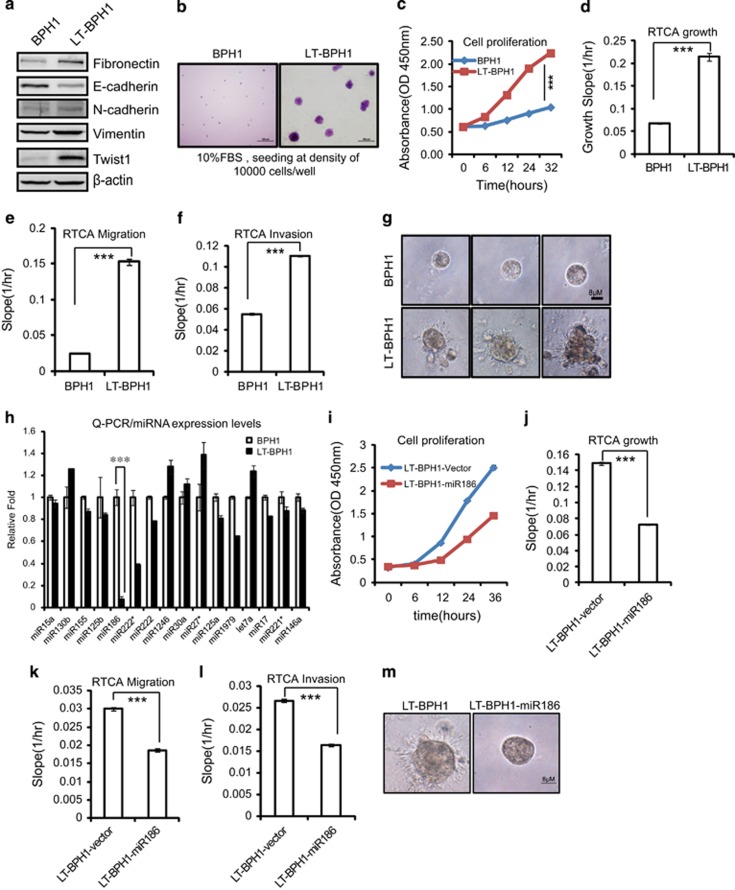
MiR186 is a key factor in inflammation-associated cell transformation. (**a**) Immunoblotting of epithelial and mesenchymal markers in BPH1 and LT-BPH1 cells. (**b**) Soft-agar colony-formation assays for BPH1 and LT-BPH1 cells. The culture medium containing 10% FBS with 0.35% agar was layered onto the base. Representative images of the colonies were shown, and the same scale bar (100 μm) was used in the images. (**c**, **i**) CCK8 cell-proliferation assays for BPH1 and LT-BPH1 cells (**c**), LT-BPH1-Vector and LT-BPH1-miR186 cells (**i**). (**d**, **j**) RTCA growth assays for BPH1 and LT-BPH1 (**d**), LT-BPH1-Vector and LT-BPH1-miR186 cells (**j**). The growth slopes were shown as histogram. Error bars indicate ±s.d., ***P-values <0.001. (**e**, **k**) RTCA migration assays for BPH1 and LT-BPH1 (**e**), LT-BPH1-Vector and LT-BPH1-miR186 cells (**k**). The migration slopes were shown as histogram. Error bars indicate ±s.d., ***P-values <0.001. (**f**, **l**) RTCA invasion assays for BPH1 and LT-BPH1 (**f**), LT-BPH1-Vector and LT-BPH1-miR186 cells (**l**). The invasion slopes were shown as histogram. Error bars indicate ±s.d., ***P-values <0.001. (**g**, **m**) Three-dimensional culture growth assays for BPH1 and LT-BPH1 (**g**), LT-BPH1-Vector and LT-BPH1-miR186 cells (**m**). Representative images of cell morphology in extracellular matrix were shown. The same scale bar (8 μm) was used in all the images. (**h**) Real-time PCR analysis for relative expression of the putative miRNAs identified by High-throughput sequencing in BPH1 and LT-BPH1 cells. U6 was used as internal control. The experiments were performed at least three independent times, and error bars indicate ±s.e.m., *P*-values of <0.001 (***).

Given that inflammatory microenvironment has key roles in tumorigenesis, we prompted to establish a chronic inflammation-driven cellular transformation model to identify regulatory circuits in PCa progression. A benign prostatic hyperplasia (BPH) epithelial cell line BPH1 was continuously treated with lipopolysaccharide (LPS; 10 μg/ml) for at least 3 weeks, which simulated chronic inflammation and sequentially caused malignant cell transformation. One of the isolated clones was designated LT-BPH1. Compared with the non-transformed parental cell line BPH1, LT-BPH1 exhibited malignantly transformed, which was further confirmed by a series of experiments, showing induced expression of maker genes of mesenchymal-like characteristics (Figure 1a) and increased abilities in anchorage-independent growth (Figure 1b), cell proliferation (Figures 1c and d and Supplementary Figure S1a), migration (Figure 1e and Supplementary Figure S1b) and invasion (Figure 1f and Supplementary Figure S1c) and disorganized growth of three-dimensional culture in extracellular matrix gels (Figure 1g). Thus, BPH1/LT-BPH1 might be regarded as an excellent model of inflammation-associated malignant transformation.

To characterize which miRNA is responsible for prostate cellular transformation, we performed the real-time PCR validation for high-throughput sequencing of miRNA profiles in BPH1 and LT-BPH1. We found that miR186 was most significantly downregulated in malignant LT-BPH1 compared with that in BPH1 (Figure 1h), which was similar to the pattern that the miR186 expression level in M12 is lower than that in P69.^25^ P69/M12 is another cellular transformation model in which M12 is a subline derived from P69 via selection in nude mice,^22^ and P69 retains some epithelial-like, low-tumorigenic and non-metastatic features while M12 displays mesenchymal-like, highly tumorigenic and metastatic characteristics.^23^ To confirm that miR186 is involved in the malignant transformation of LT-BPH1, we re-ectopically expressed miR186 in LT-BPH1 with a lentiviral expressing system,^27^ which largely reversed above their phenotypes including cell proliferation/growth (Figures 1I and j and Supplementary Figure S1d), migration (Figure 1k and Supplementary Figure S1e), invasion (Figure 1l and Supplementary Figure S1f) and three-dimensional culture growth (Figure 1m). These results indicate that miR186 may be involved in the process of malignant transformation of prostate cells. Collectively, these results demonstrated that miR186 had a critical role in inflammation-associated malignant transformation of PCa.

### MiR186 is directly activated by NF-κB under inflammation signals

**Figure 2:**
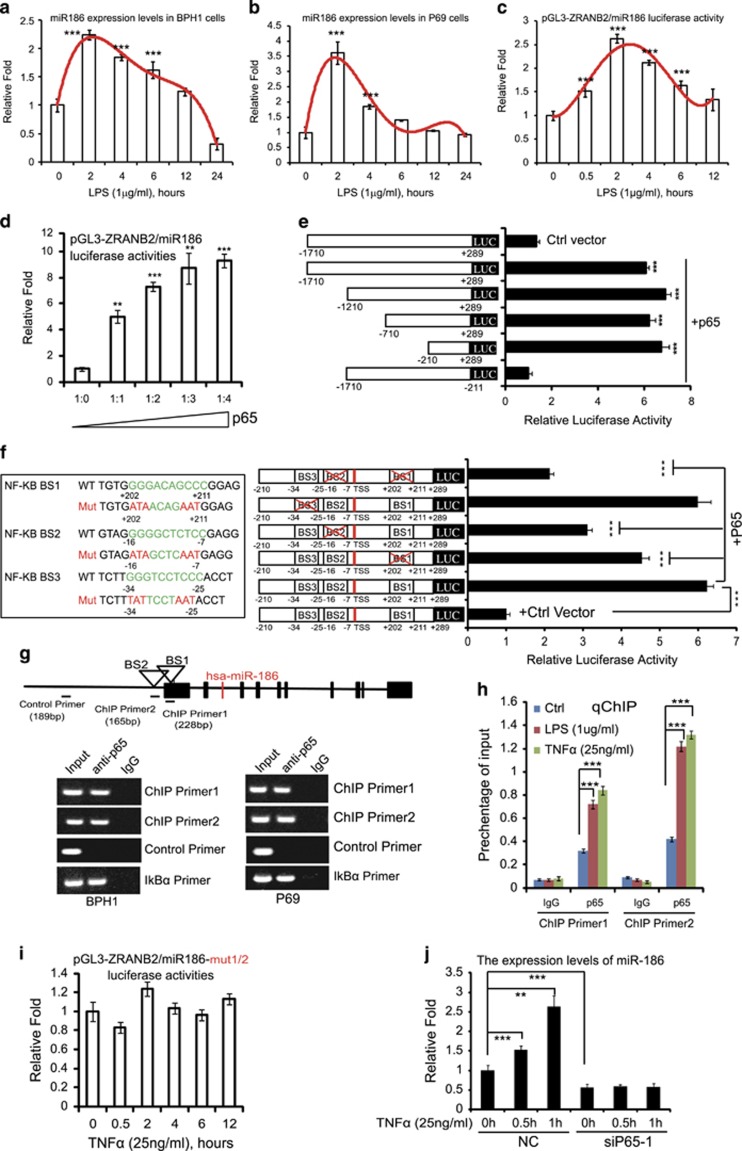
MiR186 is directly activated by NF-κB under inflammation signals. (**a**, **b**) The miR186 levels in BPH1 (**a**) and P69 (**b**) treated with LPS (1 μg/ml) for different indicated time were measured by real-time PCR. U6 was used as internal control. The experiments were performed at least three independent times, and error bars indicate ±s.e.m., ***P-values <0.001. (**c**, **d**) Luciferase activity analysis for the miR186 promoter reporter in P69 treated with LPS (1 μg/ml) for indicated different time (**c**), and in 293 T transfected with increasing amounts of NF-κB/p65 expression (**d**). The experiments were performed at least three independent times, and error bars indicate±s.e.m., **P-values <0.01 and ***P-values <0.001. (**e**) The full-length or truncated miR186 promoter reporters were introduced to 293 T together with or without NF-κB/p65 for luciferase activity analysis. The experiments were performed at least three independent times, and error bars indicate ±s.e.m., ***P-values <0.001. (**f**) Three putative NF-κB BSs in the miR186 promoter and the mutations of specific residues indicated in red (left panel); the miR186 promoter reporter constructs with single or double mutations of NF-κB BS were introduced into 293 T together with or without NF-κB/p65 for luciferase activity analysis. The experiments were performed at least three independent times, and error bars indicate ±s.e.m., *P-values <0.05, **P-values <0.01 and ***P-values <0.001 (right panel). (**g**) A schematic diagram of the ZRANB2/miR186 promoter. Hsa-miR-186 is located in the second intron of human *ZRANB2* gene; BS1 and BS2 are predicted NF-κB/p65 binding sites. The underlines indicate the fragments amplified by ChIP primer1, ChIP primer2 or control primer in ChIP analysis (top panel); ChIP analysis for p65 occupancy on BS1 and BS2 at the miR186 promoter in BPH1 and P69 (bottom panel); IκBα promoter as a positive locus. (**h**) qChIP analysis for p65 occupancy on BS1 and BS2 at the miR186 promoter in BPH1 treated with LPS (1 μg/ml) or TNFα (25 ng/ml). Data represent means±s.e.m. (*n*=3), ***P-values <0.001. The binding activity of protein is given as percentage of total input. (**i**) The luciferase activity analysis for the mutant miR186 promoter reporter containing double mutations (for NF-κB BS1 and BS2) in P69 treated with TNFα (25 ng/ml) for indicated time. The experiments were performed at least three independent times, and error bars indicate ±s.e.m. (**j**) Real-time PCR analysis for the miR186 levels in P69-Ctrl or P69-sip65-1 cells treated with TNFα (25 ng/ml) for indicated different time. U6 was used as internal control. The experiments were performed at least three independent times, and error bars indicate ±s.e.m., ***P-values <0.001.

Next, we wanted to investigate whether miR186 is involved in the inflammatory response in the induced-transformation model LT-BPH1 cells and the transformed M12 cells. As expected, miR186 in both P69 and BPH1 cells was rapidly induced in a short period of time (0–2 h) after stimulation with LPS (Figures 2a and b) or TNFα (Supplementary Figures S2a and b). To further investigate the mode of miR186 induction by LPS or TNFα, we constructed the luciferase activity reporter pGL3-ZRANB2/miR186 containing the promoter region ~2000 bp of the upstream from the transcription start site of human *ZRANB2* gene, of which the second intron harbors miR186 gene. Consistently, the luciferase activity of the ZRANB2/miR186 promoter in P69 cells was induced in a time-dependent manner upon inflammatory stimulation with LPS or TNFα (Figure 2c and Supplementary Figure S2c), suggesting that inflammatory cytokine-induced expression of miR186 was regulated at the transcriptional level.

As NF-κB activation is the most important pro-inflammatory signaling pathway, we first examined whether NF-κB/p65 is activated in BPH1 and P69 cells under treatment with TNFα or LPS. On inflammation signals, NF-κB/p65 is activated by phosphorylation on its serine 536 (S536) and subsequently translocated into nucleus. The p-S536-p65 levels in the nuclear extracts (Supplementary Figures S2d–g) were correlated with the miR186 induction in P69 and BPH1 treated with LPS or TNFα. These results indicated that p65 may be involved in the inflammation-induced miR186 expression. To validate this, the luciferase activity assay was performed by co-transfection of the reporter construct and p65, showing that p65 significantly increased the luciferase activities in a dose-dependent manner (Figure 2d). Further, to locate the NF-κB binding sites (BSs), we have constructed a series of truncated mutants of the miR186 promoter. Among them, deletion of the region −210~+289 completely abolished the reporter activity mediated by p65 (Figure 2e), revealing that the critical p65 BSs located in the region −210~+289. Three conserved p65 BSs in the region −210~+289 were predicted by using TESS (Search for Transcription Elements; Figure 2f). To pinpoint the exact BSs, we introduced point mutations into the pGL3-miR186 (−210~+289). The BS3 mutation did not affect the luciferase activity, whereas the BS1, BS2 or BS1/2 mutation reduced the activity by about 30%, 50% or 70%, respectively (Figure 2f). This result suggested that both BS1 and BS2 in the miR186 promoter were the key sites for the p65-activated transcription. The direct bindings of p65 to BS1 and BS2 in BPH1 and P69 cells were confirmed by a chromatin immunoprecipitation (ChIP) assay with two pairs of primers, primer1 for BS1 and primer2 for BS2, respectively (Figure 2g). These bindings were significantly enhanced in BPH1 cells on treatment with TNFα or LPS by qChIP (quantitative ChIP; Figure 2h). Moreover, the double mutation of BS1/2 abrogated TNFα- or LPS-mediated luciferase activity of the promoter (Figure 2i and Supplementary Figure S2h), suggesting the direct bindings of p65 to BS1/2 were required for the inflammatory induction of miR186. In addition, p65 knockdown by siRNA (Supplementary Figure S2i) prevented miR186 induction by TNFα (Figure 2j and Supplementary Figure S2i), supporting that p65 was required for miR186 induction in response to inflammatory stimulation. Collectively, these results demonstrated that the inflammatory cytokine induced a dynamic expression of miR186 in a p65-dependent manner through directly binding to the promoter of miR186.

### High methylation of CpG islands in the miR186 promoter blocks its response to inflammation signals in transformed or malignant cells

**Figure 3:**
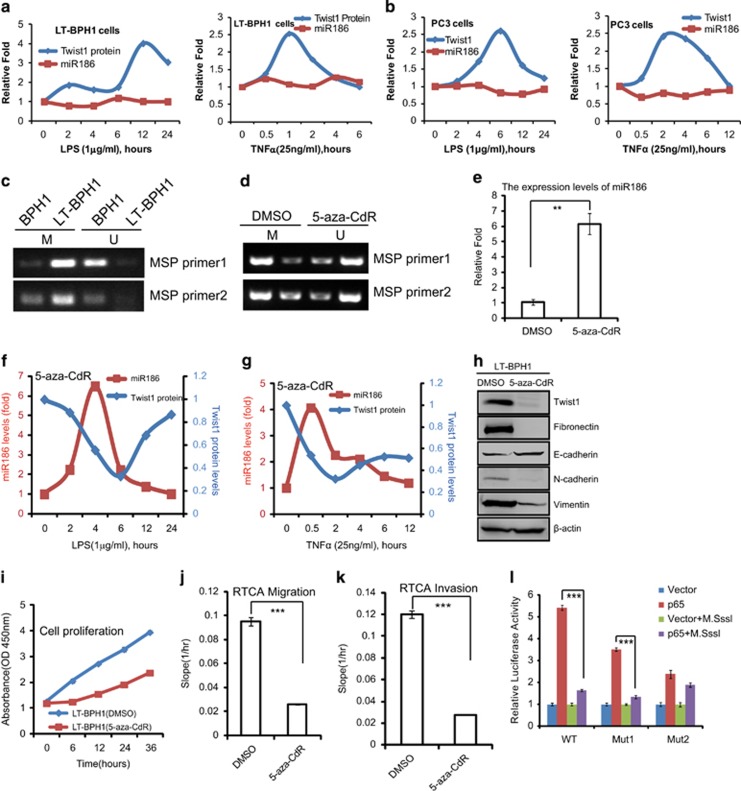
High methylation of CpG islands in the miR186 promoter blocks its response to inflammation signals in transformed or malignant cells. (**a**, **b**) LT-BPH1 (**a**) and PC3 (**b**) were treated with 1 μg/ml LPS or 25 ng/ml TNFα for different time periods as indicated, and lysed for western blotting analysis of Twist1 and real-time PCR analysis of miR186. (**c**, **d**) MSP (methylation-specific PCR) analysis for the methylation levels of CpG islands in the miR186 promoter in BPH1 and LT-BPH1 (**c**), and in LT-BPH1 treated with 5 μm 5-aza-CdR for 24 h (**d**). ‘U’ represents unmethylated DNA products amplified with non-methylation-specific primers, and ‘M’ refers to methylated DNA products amplified with methylation-specific primers. (**e**) Real-time PCR analysis for the miR186 levels in LT-BPH1 treated with or without 5 μm 5-aza-CdR for 24 h. U6 was used as internal control. The experiments were performed at least three independent times, and error bars indicate ±s.e.m., **P-values <0.01. (**f**, **g**) LT-BPH1 pretreated with or without 5 μm 5-aza-CdR for 24 h were then treated with 1 μg/ml LPS (**f**) or 25 ng/ml TNFα (**g**) for indicated different time, and lysed for western blotting analysis of Twist1 and real-time PCR analysis of miR186. (**h**) Immunoblotting of epithelial and mesenchymal markers in LT-BPH1 treated with or without 5 μm 5-aza-CdR for 24 h. (**i**) CCK8 proliferation assays for LT-BPH1 treated with or without 5 μm 5-aza-CdR for 24 h. (**j**, **k**) RTCA migration (**j**) or invasion (**k**) assays for LT-BPH1 pretreated with or without 5 μm 5-aza-CdR for 24 h, and then subjected to a dynamic migration assay lasting for 28 h (continuing addition of 5-aza-CdR to the treated cells). The migration and invasion slopes were shown as histogram. Error bars indicate ±s.d. ***P-values <0.001. (**l**) The WT, mutated NF-κB BS1 (MUT1) or mutated NF-κB BS2 (MUT2) miR186 promoter reporter was pretreated with or without M.SssI, and then co-transfected with p65 into 293 T. The relative luciferase activity was assessed. The experiments were performed at least three independent times, and error bars indicate±s.e.m., ***P-values <0.01.

As above data have revealed that NF-κB activation by LPS or TNFα can induce miR186 expression in non-transformed BPH1 and P69 cells, so we simultaneously measured the protein levels of Twist1, which is a key target of miR186 in PCa as reported in our previous study.^25^ The results showed that the rapidly induced miR186 levels were perfectly inversely correlated with downregulation of Twist1 protein in both BPH1 (Supplementary Figures S3a and b) and P69 (Supplementary Figures S3c and d) cells with the stimulation of LPS or TNFα in a time course, suggesting that NF-κB activation by LPS or TNFα was required for downregulation of Twist1, most likely via enhancing miR186 transcription. These data proposed a novel signaling pathway of NF-κB–miR186–Twist1 in BPH1 and P69 cells. However, we surprisingly observed Twist1 protein upregulation rather than downregulation in P69 cells stably transfected with anti-miR186 inhibitor for silencing endogenous miR186, under the treatment with LPS or TNFα in a time course (Supplementary Figures S3e and f). As miR186 is low expressed in the transformed LT-BPH1 (Figure 1h) or malignant PC3 (Supplementary Figure S3g), we tested the expressions of miR186 and Twist1 in these two cell lines with inflammatory stimulations. Unexpectedly, we observed miR186 was not inducible and Twist1 was increasingly induced in both LT-BPH1 (Figure 3a and Supplementary Figure S3h) and PC3 (Figure 3b and Supplementary Figure S3i) on inflammatory signals under the same treatment with LPS or TNFα in a time course. Taken together, above data suggested that the NF-κB–miR186–Twist1 axis was dependent on miR186 activation by inflammatory signals in non-transformed or low-tumorigenic cells (BPH1 and P69) rather than in transformed/malignant cells (LT-BPH1 and PC3).

Next, we attempted to explore the mechanism underlying the inactivation of the NF-κB–miR186–Twist1 pathway in transformed/malignant cells. Given the role of the promoter methylation in silencing gene expression, we wondered whether the miR186 promoter is highly methylated thus inhibiting NF-κB transcriptional activation. To address this, we first searched for CpG islands in the miR186 promoter and found a major CpG island (Supplementary Figure S4a), which is close to the TSS and contains 19 CpG dinucleotides predicted to be methylated. We found that the miR186 promoter was highly methylated in the transformed LT-BPH1 compare with that in BPH1 by using the method of methylation-specific PCR with two pairs of methylation-specific PCR primers (Figure 3c). In addition, the promoter methylation levels in malignant M12 and PC3 were higher than those in low-tumorigenic P69 (Supplementary Figure S4b). These data indicated that the hypermethylation of the miR186 promoter is probably a general phenomenon in the transformed or malignant PCa cells.

The high methylation level of the miR186 promoter in LT-BPH1 was reversed by the treatment with 5-aza-CdR, a DNA methyltransferase inhibitor (Figure 3d). Consistently with this, the miR186 expression was obviously increased by about sixfold under the treatment with 5-aza- CdR (Figure 3e). More importantly, we observed that the pretreatment of 5-aza-CdR restored miR186 responsiveness to LPS (Figure 3f and Supplementary Figure S4c) and TNFα (Figure 3g and Supplementary Figure S4d) and subsequently inhibiting the Twist1 expression in LT-BPH1 cells, which suggested that miR186 expression was strictly controlled by the methylation level of its promoter. Furthermore, the phenotypes of LT-BPH1 were also reversed by treatment with 5-aza-CdR, showing that EMT progression (Figure 3h), cell proliferation (Figure 3i), migration (Figure 3j and Supplementary Figure S4e) and invasion (Figure 3k and Supplementary Figure S4f) were attenuated. In addition, we did not observe that the CpG methylation levels of the Twist1 promoter were different between BPH1 and LT-BPH1, even in both these cells treated with 5-aza-dC, and among P69, M12 and PC3 cells (Supplementary Figure S4g). These excluded the possibility that the methylation of CpG islands in the Twist1 promoter was involved in the above events.

To further figure out whether the CpG methyaltion is connected with the NF-κB-mediated transcriptional activation, the pGL3-miR186 (−210~+289) encompassing p65 BSs and CpG islands was premethylated *in vitro* with a CpG methyltransferase M.SssI (specifically methylates CpG dinucleotides), and then transfected into 293T cells. As expected, the *in vitro* CpG methylation almost abrogated the luciferase activity of the wild-type or p65-BS1 mutated promoter induced by p65, whereas the mutation of p65-BS2 did not significantly affect (Figure 3l). These results suggested that the CpG methylation of miR186 promoter was involved in the p65-mediated transcription activity, and the p65-BS2 was sensitive to the CpG methylation. Collectively, our results demonstrated that the hypermethylation of the miR186 promoter was responsible for its downregulation or blocking p65 transcription activity, thus no responsiveness to inflammatory signals in the transformed or malignant PCa cells.

### Twist1 represses miR186 in a negative feedback loop through directly interacting with and recruiting Dnmt3a to the miR186 promoter

**Figure 4:**
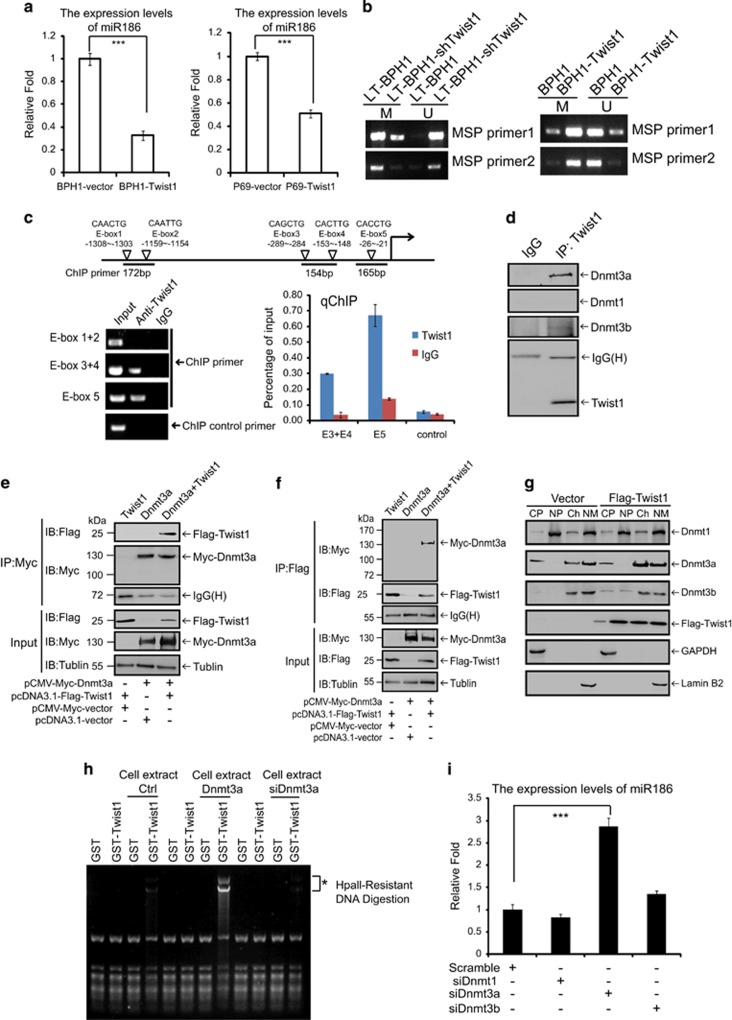
Twist1 represses miR186 in a negative feedback loop through directly interacting with and recruiting Dnmt3a to the miR186 promoter. (**a**) Real-time PCR analysis for the relative expression of miR186 in BPH1-Vector and BPH1-Twist1, or P69-Vector and P69-Twist1 cells. ***P-values <0.001. (**b**) The methylation-specific PCR (MSP) analysis for the methylation levels of CpG islands at the miR186 promoter in LT-BPH1 and LT-BPH1-shTwist1 or BPH1 and BPH1-Twist1. (**c**) A schematic diagram of Twist1 binding E-boxes mapped at the miR186 promoter. The fragments amplified in ChIP or qChIP are indicated (top panel); ChIP or qChIP analysis for Twist1 occupancy on E-box3/4, E-box5 at the miR186 promoter (bottom panel). (**d**) Lysates from LT-BPH1 were used for co-IP with anti-Twist1 antibody or anti-IgG, and followed by immunoblotting with antibodies against Dnmt3a, Dnmt3b, Dnmt1 and Twist1. (**e**, **f**) 293 T cells transfected with Flag-Twist1 and Myc-Dnmt3a were lysed for co-IP with anti-Myc (**e**) or anti-Flag (**f**) antibody, and followed by western blotting with antibodies anti-Myc and anti-Flag. Lysates as input were immunoblotted with antibodies anti-Myc, anti-Flag and anti-Tubulin. (**g**) Western blotting analysis for 293 T transfected with the control Vector or Flag-Twist1 were fractionated into nucleoplasm (NP), chromatin (Ch), cytoplasm(CP) and nuclear matrix (NM), and probed with anti-Flag, anti-Dnmt1/3a/3b or anti-Lamin B2 antibodies. Lamin B was a control for NM-associated protein. GAPDH is used as a control for cytoplasmic protein. (**h**) Purified GST or GST-Twist1 proteins and the miR186 promoter construct were mixed with different extracts of 293 T transfected with control Vecter, Dnmt3a or siDnmt3a, respectively, 2 h after incubation, followed by DNA digestion with HpaII. As negative controls, GST and GST-Twist1 without incubation of cell extracts did not show any intrinsic DNA methyltransferase activity. Methylated bands are indicated by an asterisk (*). (**i**) Real-time PCR analysis for the relative expression of miR186 in BPH1 cells transfected with scrambled siRNA, siDnmt1, siDnmt3a or siDnmt3b.

To better understand the relationship between miR186 and Twist1, we stably overexpressed Twist1 in P69 and BPH1 cells and found the expression levels of miR186 were remarkably decreased (Figure 4a), which indicated that miR186 and Twist1 probably constituted a negative regulatory feedback loop. Most interestingly, the methylation-specific PCR assays revealed that stable overexpression or knockdown of Twist1 significantly increased or decreased the methylation levels of the miR186 promoter, respectively (Figure 4b). This suggested that Twist1 was involved in the regulation of the miR186 promoter methylation level. Twist1 is characterized by a basic DNA-binding domain that targets the consensus E-box sequence ‘5-CANNTG-3’. To reveal how Twist1 negatively regulates miR186, we first searched the E-boxes in the miR186 promoter and found multiple E-boxes adjacent to TSS. The luciferase activity assays showed that Twist1 repressed the miR186 promoter activity in a dose-dependent manner (Supplementary Figure S5a). The ChIP and qChIP assays further demonstrated that Twist1 directly interacted with the miR186 promoter at E-boxes 3, 4 and 5, rather than E-boxes 1, 2 in LT-BPH1 cells (Figure 4c). As Twist1 lacks the methyltransferase activity, it may recruit some partners like methyltransferases for methylation. To address this, we investigated the interaction between Twist1 and cellular DNA methyltransferases, and showed that endogenous Twist1 strongly bound to Dnmt3a, weakly to Dnmt3b but not to Dnmt1 in LT-BPH1 (Figure 4d). The binding of Twist1 with Dnmt3a in LT-BPH1 cells was also confirmed by in a reciprocal co-IP assay (Supplementary Figure S5b). The strong interaction between Flag-Twist1 and Myc-Dnmt3a exogenously expressed in 293T was also validated (Figures 4e and f). To further test whether Twist1 directly binds to Dnmt3a, we performed the GST pull-down assay to show that GST-Twist1 could bind to Dnmt3a *in vitro* (Supplementary Figure S5c). Next, to map which region on Twist1 or Dnmt3a protein is required for their interaction, we constructed the GST-Twist1-N(1–107 aa) and GST-Twist1-C(108–203 aa) and found the N terminus (1–107 aa) of Twist1 was necessary for the binding with Dnmt3a (Supplementary Figure S5d). We also generated the truncated or deleted construct forms of Dnmt3a, and found both D2 (476–912 aa) and D4 (627–912 aa) of Dnmt3a could not bind to GST-Twist1, whereas both D1 (1–476 aa) and D3 (1–627 aa) strongly bound to GST-Twist1 (Supplementary Figure S5e). Although both truncated forms D1 (only containing PWWP domain) and D3 (containing PWWP and ADD domains) strongly bound to GST-Twist1, the D2 also containing ADD domain could not bind with GST-Twist1, which suggested that ADD domain was dispensable for the binding with Twist1, while PWWP domain in D1 of Dnmt3a was probably sufficient for the binding with Twist1.

Although above results showed that Twist1 directly interacted with Dnmt3a in cells, it is unknown whether Twist1 recruits Dnmt3a to chromatin. Therefore, we extracted the cytoplasm (CP), nucleoplasm (NP), chromatin (Ch) and nuclear matrix (NM) and examined the compartmentalization of Dnmt3a. Stable overexpression of Twist1 dramatically increased the distribution of Dnmt3a but not Dnmt3b in the chromatin (Figure 4g). Next, to test whether Twist1-associated Dnmt3a is *de novo* methyltransferase-active, GST-Twist1 and the miR186 promoter construct were incubated with different extracts of cells transfected with the control Vecter, Dnmt3a or siDnmt3a, and followed by DNA digestion with HpaII, a methylation-resistant restriction enzyme. After GST-Twist1 was incubated with cell extracts from the control Vector-transfected cells, HpaII-resistant DNA fragments of the miR186 promoter was observed. These HpaII-resistant fragments were strengthened in GST-Twist1 incubated with cell extracts from the Dnmt3a-overexpressing cells, whereas they were greatly weakened in GST-Twist1 incubated with cell extracts from the Dnmt3a-silencing cells (Figure 4h), which suggested that Twist1 recruited Dnmt3a from cell extracts to *de novo* methylate the miR186 promoter. Moreover, to confirm whether the Dnmt3a is indeed involved in the miR186 expression through methylating its promoter, we knocked down DNMTs by siRNAs in BPH1 cells and found that only knockdown of Dnmt3a, but not of either Dnmt1 or Dnmt3b, significantly increased the miR186 levels (Figure 4i). Thus, the above results elucidated a negative feedback loop of Twist1 regulating the miR186 expression through directly recruiting an active Dnmt3a to the miR186 promoter, which facilitated methylation in turn repressing miR186 expression.

### Twist1–Dnmt3a complex facilitates the site-specific CpG methylation of the miR186 promoter to block the transcriptional activity of NF-κB

To investigate the mechanism that the CpG methylation by Twist1-recruited Dnmt3a blocks miR186 responsiveness to inflammatory signals, we analyzed the miR186 promoter and found that the NF-κB BSs, Twist1 BS/E-Box and CpG inlands all tandem-gathered in the short region from −25 to approximately −6 bp: Twist1 BS/E-Box5 (−25 to approximately −20 bp), NF-κB BS2 (−16 to approximately −7 bp) and CpG8 (−7 to approximately −6 bp; Supplementary Figure S6a). These indicated a fine-tuned regulatory mechanism of Twist1–Dnmt3a–^m^CpG-NF-κB axis on the miR186 expression.

**Figure 5:**
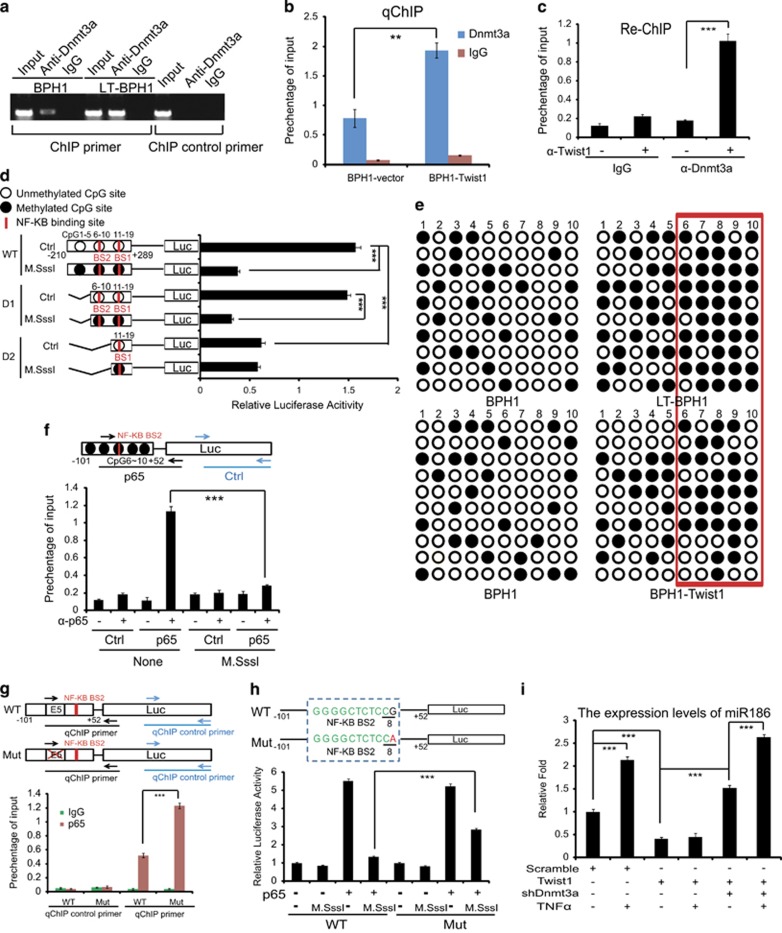
Twist1 recruiting Dnmt3a facilitates the site-specific CpG methylation of the miR186 promoter to decrease the binding and the transcriptional activity of NF-κB/P65. (**a**) ChIP analysis for Dnmt3a occupancy at miR186 promoter in BPH1 and LT-BPH1. (**b**) qChIP analysis for Dnmt3a occupancy at the miR186 promoter in BPH1 and BPH1-Twist1 cells. Data represent means±s.e.m. (*n*=3), **P-values <0.01. The binding activity of protein is given as percentage of total input. (**c**) LT-BPH1 lysates were subjected to ChIP with anti-Dnmt3a antibody, and followed by re-ChIP with anti-Twist1 antibody. The DNA fragments were analyzed by qPCR. (**d**) The full-length or truncated miR186 promoter reporters were *in vitro* premethylated with M.Sssl, and then co-transfected with NF-κB/p65 into 293 T for the luciferase activity assays. The experiments were performed at least three independent times, and error bars indicate ±s.e.m., ***P-values <0.01. (**e**) Bisulfite sequencing to determine the methylation status of the critical miR186 promoter region in BPH1 and LT-BPH1 (top panel), or BPH1 and BPH1-Twist1 (bottom panel). ○, nonmethylated cytosine; •, methylated cytosine; red box, CpG6~10. (**f**) The minimal miR186 promoter (−101~+52) containing the CpG6~10 and the NF-κB BS2 were premethylated *in vitro* by M.Sssl before co-transfection with the construct p65 in 293 T. qChIP assay was performed with anti-p65 and qPCR primers specific for the p65 region ‘—(black)’ or nonspecific region control ‘— (blue)’. The experiments were performed at least three independent times, and error bars indicate ±s.e.m., *P-values <0.05, **P-values <0.01 and ***P-values <0.001. (**g**) A schematic representation of the minimal miR186 promoter (−101~+52) containing NF-κB BS2 with WT or mutant E-box5 (E5). The arrows indicate the fragment amplified in qChIP analysis (top panel); qChIP analysis for p65 occupancy at the above two constructs of the minimal miR186 promoter in 293 T. Data represent means±s.e.m. (*n*=3), ***P-values <0.001. The binding activity of protein is given as percentage of total input (bottom panel). (**h**) A schematic representation of the minimal miR186 promoter (−101~+52) containing the WT-CpG8 or mut-CpA used for the luciferase activity assay (top panel); the indicated constructs were or not premethylated *in vitro* by M.SssI, and then co-transfected with or without the p65 expression construct in 293 T for the luciferase activity assay. The experiments were performed at least three independent times, and error bars indicate ±s.e.m., *P-values <0.05, **P-values <0.01 and ***P-values<0.001 (bottom panel). (**i**) Real-time PCR analysis for the miR186 levels in BPH1 cells stably transfected with or without Twist1 and shDnmt3a, or treatment of TNFα (25 ng/ml) for 4 h. The experiments were performed at least three independent times, and error bars indicate ±s.e.m., ***P-values <0.001.

To reveal whether Dnmt3a directly binds to the miR186 promoter, we performed a ChIP assay and showed that Dnmt3a indeed bound to the promoter CpG inland region and this binding in LT-BPH1 was much stronger than that in BPH1 (Figure 5a), which could explain for the hypermethylation of miR186 promoter in LT-BPH1. To confirm that Twist1 interacting with Dnmt3a is required for the latter binding to the specific CpG island, we ectopically expressed or knocked down Twist1 in BPH1 and found that the binding of Dnmt3a to the CpG inlands in the miR186 promoter was significantly increased or decreased, respectively (Figure 5b and Supplementary Figure S6b). Moreover, we performed a Re-ChIP assay in LT-BPH1 and revealed that Dnmt3a and Twist1 cooperatively bound to the CpG island region of the miR186 promoter (Figure 5c and Supplementary Figure S6c). These data strongly suggested that Twist1 promoted the association of Dnmt3a with the CpG inlands at the miR186 promoter.

To further pinpoint the exact CpG that is critical for NF-κB-dependent miR186 expression, the luciferase reporter assays for the full-length or truncated miR186 promoters premethylated by M.SssI *in vitro* were performed. The results showed that the premethylation dramatically reduced the luciferase activity of the full-length (containing CpG 1–19) and D1 (containing CpG 6–19) but not D2 (containing CpG 11–19) miR186 promoter, indicating that the reduction of miR186 promoter activity was mainly dependent on the methylation of CpG 6~10 island, which is overlapping or around the NF-κB BS2 (Figure 5d). Furthermore, the bisulfite sequencing results showed that the methylation levels in the miR186 promoter, especially the region CpG6~10, which is overlapping or around the p65-BS2, were much higher in LT-BPH1 than BPH1 (Figure 5e, upper panels). The methylation levels of CpG6~10 were obviously increased when Twist1 was overexpressed in BPH1, which indicated that Twist1 was involved in the site-specific methylation of CpG6~10 in the miR186 promoter (Figure 5e, lower panels).

To investigate the CpG methylation-mediated suppression of the miR186 promoter activity, the miR186 minimal promoter construct containing the NF-κB BS2 and CpG 6~10 was premethylated *in vitro* with M.SssI and then co-transfected with p65 into 293T, followed by qChIP assay. The results showed that pretreated methylation with M.Sssl significantly reduced the binding of NF-κB to the minimal miR186 promoter compared with that in the untreated group (Figure 5f), which revealed that methylation especially at the CpG 6~10 islands effectively abrogates the NF-κB transcription activity on the miR186 promoter through interfering with the binding of NF-κB to BS2. As Twist1 E-box5 is the closest to NF-κB BS2 and located in the region of CpG 6–10 islands (Supplementary Figure S6a), we wondered whether the binding of Twist1 to E-box5 prevents NF-κB from interacting with BS2, which is essential for the miR186 promoter activation and sensitive to CpG methylation. Thus, we showed the mutation of E-box5 significantly increased the affinity of NF-κB binding to the miR186 promoter (Figure 5g). Furthermore, we performed the same experiment as in Figure 5d, and showed that the reduction of the luciferase activity of the wild-type minimal miR186 promoter by M.Sssl premethylation was partially recovered by mutating ‘G’ of CpG8 to ‘A’, suggesting that CpG8 is a key methylation site for inhibition of NF-κB binding to BS2 and transcriptional activity (Figure 5h). Last, we confirmed that the TNFα-induced miR186 expression was abolished by ectopically expressing Twist1 in BPH1, which was rescued by knockdown of Dnnmt3a, suggesting that the association of Twist1 with E-box5 could inhibit the binding of NF-κB to BS2 and its transcriptional activity of the miR186 promoter in a Dnmt3a-dependent manner (Figure 5i). Taken together, these results demonstrated that Twist1 recruited Dnmt3a and consequently facilitated the site-specific CpG methylation to decrease the binding and the transcriptional activity of NF-κB on the miR186 promoter.

### The miR186 expression and its promoter methylation are related with inflammation-associated human PCa

**Figure 6:**
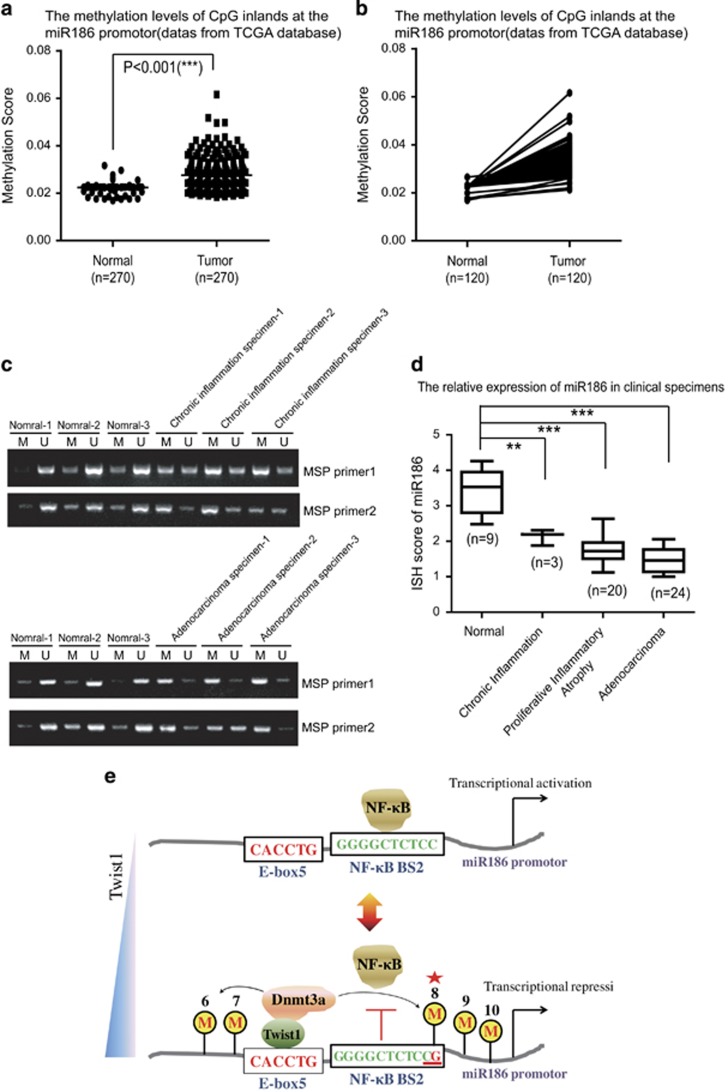
The miR186 expression and its promoter methylation are related with inflammation-associated PCa. (**a**) Methylation scores of miR186 promoter were derived from the TCGA database, including prostate cancer samples (*n*=270) and normal samples (*n*=270). (**b**) Comparison of the methylation levels of miR186 promoter in the top 120 most significant difference paired clinical cases. The dots represent the relative methylation score of miR186 promoter and each pair of dots connected by a dash line denotes a set of paired specimens. (**c**) DNA isolated from normal, chronic inflammation or tumor samples was subjected to methylation-specific PCR using methylated-specific (‘M’) and unmethylated-specific (‘U’) primers from the miR186 promoter. (**d**) Assessment of miR186 levels by *in situ* hybridization in normal prostate tissues, chronic inflammation specimens, proliferative inflammatory atrophy tissues and adenocarcinoma specimens. (**e**) A schematic representation of the proposed Twist1-dependent site-specific CpG8 methylation through recruiting Dnmt3a to the miR186 promoter to block the transcriptional activity of NF-κB/p65.

Further to investigate whether the methylation levels of the miR186 promoter is related with the miR186 expression level in the clinical inflammation-associated human PCa specimens, we analyzed the miR186 promoter methylations in PCa and normal tissues across a large number of PCa samples (*n*=270) and normal controls (*n*=270) through the TCGA database (The Cancer Genome Atlas). The results revealed significant increases in the methylation levels of CpG inlands at the miR186 promotor in PCa samples compared with those in normal tissues (Figure 6a). In particular, the top 120 most significant difference paired clinical cases of them were selected for comparison analysis to show that the methylation levels of the miR186 promoter in the PCa tissues was higher than those in normal tissues (Figure 6b). Moreover, the methylation-specific PCR analyses with three pairs of normal tissue specimens and chronic inflammation specimens or adenocarcinoma specimens were performed to show that the miR186 promoter was highly methylated in all chronic inflammation and tumor tissues compared with that in normal tissues (Figure 6c). More interestingly, the *in situ* hybridization analysis revealed that the expression levels of miR186 were reduced not only in the tumor tissues, but also in the chronic inflammation and proliferative inflammatory atrophy tissues, which is a lesion that links inflammation and PCa. The miR186 expression levels in the prostate tissues were normal > chronic inflammation > proliferative inflammatory atrophy > adenocarcinoma (Figure 6d). Collectively, these results suggested that the miR186 promotor methylation was correlated with inflammation-associated PCa progression and decreased miR186 likely contributed to inflammation–cancer transformation.

## Discussion

PCa is the most common malignant tumor in men and the second highest cause of cancer mortality in developed western countries.^1, 28^ Increasing evidences suggest that the inflammatory microenvironment has a key role in PCa progression.^29^ Numerous epidemiologic survey and meta-analysis have shown that there is a significant increase in the relative risk of PCa in men with prostatitis.^30, 31, 32^ Histologically, most lesions that contain chronic inflammatory infiltrates in the prostate are associated with atrophic epithelium or focal epithelial atrophy, especially the proliferative inflammatory atrophy, which is regarded as the link between inflammation and PCa.^33^ However, the underlying mechanisms between inflammation and PCa remain largely unknown. Here, we described an inflammatory signaling feedback loop involving NF-κB/p65, miR186, Twist1 and Dnmt3acontrolled PCa development through epigenetic switch, which was responsible for maintenance of the malignantly transformed state. Understanding the dynamic regulatory circuitry of signal transduction in PCa could pave the way to new therapeutic approaches and personalized treatments.

### MiR186 is an inflammatory response gene

Our data showed that miR186 was rapidly induced through the direct binding of activated NF-κB/p65 to the miR186 promoter in P69 and BPH1 cells under inflammatory stimuli (LPS, TNFα), suggesting that miR186 was a new inflammatory response gene except for miR155^16^ and miR21,^18^ which are regulated by inflammation signaling, linking inflammation to prostate carcinogenesis. The inflammation-induced miR186 expression was presumably a general response in the non-tumorigenic, non-transformed cell lines but not in the transformed or malignant prostate cancer cell lines (LT-BPH1, M12 or PC3).

### Twist1 drives PCa tumorigenesis and metastasis

We observed that miR186 overexpression significantly decreases the Twist1 protein level in LT-BPH1, M12 and PC3, which provided crucial insight into miR186 executing tumor-suppressing function by directly targeting Twist1 in malignant PCa cells. Twist1 participates in many cellular processes such as proliferation, EMT, invasion/metastasis,^34, 35, 36, 37^ angiogenesis/VM,^38, 39^ cancer stem cell formation,^40^ multi-drug resistance^41^ and apoptosis inhibition.^42^ Twist1 is highly expressed in many different types of aggressive tumors, especially PCa.^24, 43, 44^ Our data provided evidences that the high Twist1 level played extremely important roles in driving cancer initiation, progression and metastasis in PCa. In addition to being a biomarker of advanced PCa, an approach with reducing Twist1 to the physiological level is sufficient for anticancer effects,^45^ so inhibition of Twist1 may be a potential novel therapeutic strategy for PCa treatment.

### NF-κB–miR186–Twist1 versus NF-κB–Twist1

The NF-κB pathway is thought to have a role in oncogenic function; however, in our study, we revealed that NF-κB acted as a tumor suppressor by inducing miR186 expression and subsequently downregulating Twist1 expression in BPH1 and P69 under inflammatory stimulation. On the contrary, we also observed that NF-κB directly upregulated Twist1 expression through binding to the promoter^13^ in LT-BPH1, M12 and PC3 with treatment of LPS or TNFα. And simultaneously, miR186 was not able to be induced, indicating that the pathway of miR186-mediated Twist1 downregulation was blocked in those transformed or malignant PCa cells. The antagonism of these pathways kept the dynamic expression of Twist1, and their fluctuation resulted in distinct consequence of tumor suppression or promotion. According to these results, we proposed that the Twist1 protein level was fine-tuned by two signaling pathways; one was a known oncogenic signaling pathway NF-κB–Twist1, whereas the other was a new identified tumor-suppressing pathway NF-κB–miR186–Twist1. Twist1 was downregulated by the NF-κB–miR186–Twist1 pathway in non-transformed and normal cells (BPH1, P69), whereas this pathway was abolished due to the CpG hypermethylation of the miR186 promoter in the malignantly transformed and cancer cells (LT-BPH1, M12, PC3), thereby losing antagonization of Twist1 upregulation from the NF-κB–Twist1 pathway, which was still active. Thus, the above unbalance led to the high Twist1 level and subsequently many EMT-related, oncogenic and metastatic genes were transcribed and translated, thereafter promoting malignant transformation, tumorigenesis and metastasis.

### Twist1 recruiting Dnmt3a to mediate the site-specific CpG methylation

Twist1 is highly expressed in 90% of PCa tissues and positively associated with PCa Gleason grading,^24^ indicating that Twist1 has an important role in PCa progression. Twist1 can recruit some chromatin-modification related complexes to change the chromatin structure. For examples, Twist1 recruits Mi2/NuRD complex including HDAC1 and HDAC2 to the E-cadherin and ERα promoters causing histone deacetylation and chromatin condensation, further reducing transcript levels, thus to promote EMT and metastasis of breast cancers.^46, 47^

The mechanism underlying miR186 lowly expressed in PCa remains unknown. Here, we observed that losing of miR186 induction under inflammatory stimuli in malignancy was connected to the promoter methylation. This regulation involved the site-specific CpG methylation, which blocked NF-κB binding to its BSs at the miR186 promoter. We found that the CpG islands adjacent to the NF-κB BS2 were hypermethylated and NF-κB was no longer able to associate with the miR186 promoter, which resulted in repression of miR186 transcription. We also found that only Dnmt3a, but not Dnmt3b, mediated methylation of the miR186 promoter. Twist1 lacking enzymatic activity interacted with Dnmt3a *in vivo* and *in vitro*, and this interaction re-localized Dnmt3a from the nuclear matrix into chromatin, specifically increasing the association of Dnmt3a with the miR186 promoter.

### A double-negative feedback regulatory circuit of NF-κB–miR186–Twist1–Dnmt3a

We further demonstrated that the regulatory mechanism of Twist1/miR186 expression was a double-negative feedback regulatory circuit: Twist1, a downstream target of miR186, negatively repressed miR186 expression by increasing Dnmt3a-mediated CpG methylation of the miR186 promoter, which abolished the NF-κB-dependent enhancement of miR186 transcription in malignant cells. Our results strongly pointed out that the very low expression of miR186 in the transformed or malignant PCa cells was mainly due to the hypermethylation of miR186 promoter to block the responsiveness to the inflammatory signals and NF-κB transcription activity.

We found that Dnmt3a itself with no substrate specificity was recruited for the site-specific CpG methylation on the miR186 promotor by Twist1, which efficiently bound to the E-box motif at the miR186 promotor. In particular, the site-specific methylation of CpG (CpG6~10) region containing NF-κB BS2 blocked the binding and transcription activity of NF-κB on the miR186 promoter. It is noted that methylation of a single nucleotide C can be sufficient for repression of the promoter activation,^48^ and we finally identified that methylation of CpG8 by Twist1–Dnmt3a complex was required and sufficient for blocking the binding of NF-κB to BS2, which led to repressing the miR186 expression (Figure 6e).

In summary, we proposed a double-negative feedback regulatory circuit controls inflammation-associated prostate cancer progression (Supplementary Figure S7). The high level of Twist1 triggered this feedback loop underlying the epigenetic switch, which was essential for maintaining transformed and advanced state of PCa. This model might explain the epigenetic switch from non-transformed to transformed, in which the high Twist1 level constituted the feedback loop to inhibit the transcriptional activation of miR186 induced by NF-κB. Most importantly, once this feedback was activated, the regulatory circuit was sufficient to generate and maintain the transformed state and cancer cell malignancy.

## Materials and methods

### Establishment of an inflammation-associated transformed cell line LT-BPH1

BPH1 cells were continuously exposed to LPS (10 μg/ml) for at least 3 weeks to establish chronic inflammatory cell lines. To isolate a malignantly transformed clone, a series of experiments were performed including EMT marker protein changes by western blotting, soft-agar colony-formation assays, cell proliferation by RTCA (real-time cell analysis), cell migration and invasion by RTCA, three-dimensional culture assays. Compared with the non-transformed parental cell line BPH1, a malignantly transformed cell line was selected and designed LT-BPH1. Thus, BPH1 and LT-BPH1 is a pair of excellent prostate cell lines for study on the molecular mechanisms underlying chronic inflammation promoting malignant transformation of cells and carcinogenesis.

### Cell fractionation

Nuclear matrix fractionation and chromatin fractionation was performed as described.^49^

### RTCA migration and RTCA invasion

The cell migration and invasion experiments were performed using the xCELLigence RTCA DP system (Roche, Mannheim, Germany) as described previously.^23, 50^ Briefly, cells serum-starved for 6 h were resuspended with serum-free medium and seed into up chamber of CIM-plate 16 with or without precoated Matrigel (1:40 dilution), which the low chamber was added medium containing 5% FBS, for real-time cell analysis by using the RTCA DP Instrument. The increase of the impedance correlates with increasing numbers of migrated cells were recorded every 15 min and the cell index Slope (1/hour) for a time period/range was calculated according to RTCA Software Manual (Version 1.2).

### *In vitro* methylation of the miR186 promoter

The indicated miR186 reporter constructs were methylated by incubation with M.SssI methylase (New England BioLabs, Beijing, China) for 3 h at 37 °C in the presence of 160 mm S-adenosylmethionine. The methylation status was verified by digestion with HhaI, a methylation-sensitive enzyme.

### Clinical prostate cancer specimens

Clinical prostate cancer specimens and prostate cancer tissue arrays were purchased from ALenabio (Catalog #PR8011a, Xi’an, China).

### Statistical analysis

All statistical analyses were carried out using the SPSS 18.0 statistical software package (SPSS, Chicago, IL, USA). All the experiments were repeated three times and all the data were presented as mean±s.e.m. Comparisons between groups for statistical significance were conducted with a two-tailed Student’s *t*-test. The differences between multiple groups were analyzed by one-way analysis of variance. *P<0.05, **P<0.01 and ***P<0.001 were considered statistically significant in all the cases.
