# Supplementary material for: Twist1/Dnmt3a and miR186 establish a regulatory circuit that controls inflammation-associated prostate cancer progression
Source: Oncogenesis. 2017 Apr 10;6(4):e315–. doi: 10.1038/oncsis.2017.16 (PMC5520493; doi:10.1038/oncsis.2017.16)
Supplement: Supplementary Information [file oncsis201716x1.docx]

**SUPPLEMENTARY METHODS**

**Cell lines and culture**

Human **b**enign **p**rostatic **h**yperplasia epithelial cell line BPH-1[^4^](#_ENREF_4) is a gift from Dr. Simon W. Hayward, Vanderbilt University Medical Center, USA. BPH-1 and prostate cancer cell line PC3 were cultured in RPMI 1640 (Hyclone, Logan, UT, USA) containing 10% FBS, 100 U/ml penicillin and 100 mg/ml streptomycin. HeLa and HEK293T cells were cultured in DMEM (Hyclone) growth medium supplemented with antibiotics and 10% FBS. P69 is a SV40-T antigen-immortalized human prostate epithelial cell line which is low-tumorigenic and non-metastatic,[^1^](#_ENREF_1) while M12 is a highly tumorigenic and metastatic subline of P69 cells generated by selection in nude mice.[^2^](#_ENREF_2)^,^ [^7^](#_ENREF_7) Both cell lines were cultured in RPMI 1640 supplemented with 5% fetal bovine serum (FBS), 10 ng/ml epidermal growth factor (EGF), 0.1 μM dexamethasone, 5 μg/ml insulin, 5 μg/ml transferrin, 5 ng/ml selenium and 0.05 mg/ml gentamicin. Cultures were maintained at 37°C in a 5% CO_2_ cell incubator. All cell lines have been tested for mycoplasma contamination before use.

**Vasculogenic mimicry and 3D culture assays**

Cells were allowed to grow to 80% confluence of monolayer. For vasculogenic mimicry assay[^8^](#_ENREF_8), matrigel matrix^TM^ (#3445-005-01,Trevigen, Gaithersburg, MD, USA) pre-thawed at 4°C were added into the inner well of μ-slides (ibidi Gmbh, Martinsried, Germany) and incubated for at least 30 min at 37°C until polymerization. 50 μl of cells (1×10^5^ cells/ml) were added onto the polymerizd matrix. For 3D culture assays for invasion, [^7^](#_ENREF_7)5 μl of cells (1×10^5^ cells/ml) mixed with 5 μl of 3D culture matrix^TM^ were added into the inner well of μ-slides and incubated for at least 60 min at 37°C. After polymerization, cell-free medium were added to fill the upper well. Microscopy images were taken after three days or longer time.

**microRNA and mRNA quantification by real-time PCR**

This method was described as before.[^6^](#_ENREF_6) Briefly, total RNA was extracted using Trizol reagent (Invitrogen, CA, USA). 1μg of each sample was used for reverse transcription by AMV Reverse Transcriptase (Fermentas, CA, USA), according to the manufacturer’s instructions. Speciﬁc stem-loop reverse transcription primers for miRNAs were designed as described.[^3^](#_ENREF_3) The miRNA levels were analyzed using the SYBR-Green Master PCR Mix (Applied Biosystems) with an ABI Stepone system (Applied Biosystems, Foster City, CA). The amplification parameters were 95 °C for 10 min followed by 40 cycles of 95 °C for 15 s, 55 °C for 30s and 72 °C for 30s. The expression of miRNA were normalized and expressed as a percentage relative to U6 respectively with the following formula: fold induction = 2^[−ΔCt]^, where ΔCt = Ct_(target)_ − Ct_(U6 or GAPDH)_. Each sample was analyzed in at least triplicate. The following primer sequences were used:

miR186 reverse transcription primer: GTCGTATCCAGTGCAGGGTCCGAGGTATTCGCACTGGATACGACAGCCCA;

miR186 forward: GCCGGCAAAGAATTCTCCTTT,

common miRNA reverse: GTGCAGGGTCCGAGGT;

U6 forward: CTCGCTTCGGCAGCACA;

U6 reverse primer: AACGCTTCACGAATTTGCGT.

**Dual luciferase reporter assays**

For measurement of NF-κB/p65 targeting the Twist1 promoter, 293T cells (5×10^4^ cells per well) were plated in a 24-well plate and then co-transfected with 200 ng of either pcDNA3.1-Flag-P65 or pcDNA3.1 as a control, 100 ng of either pGL3-Basic (Promega) Fireﬂy luciferase constructs containing wild-type or mutated ZRANB2/miR186 promoter regions, and 20ng of pRL-CMV Renilla Luciferase vector as a normalization control using Lipofectamine^TM^ 2000. 293T cells were collected 48 h after transfection and analyzed using the dual-luciferase reporter assay system. The pRL-CMV vector that shows constitutive expression of Renilla luciferase was co-transfected as an internal control to correct for differences in transfection. Transfections were performed in triplicate and repeated at least three independent times.

**Soft-agar colony formation assays**

The ability of anchorage-independent growth was evaluated by a soft agar assay as described.[^5^](#_ENREF_5) Briefly, 3×10^3^ cells of each clone were suspended in culture medium containing 5% or 2% FBS with 0.35% Bacto agar (Amresco, OH, USA). The agar cell mixture was plated on top of a bottom layer with 0.6% agar-medium mixture in six-well plates. After 14 ~18 days, cell colonies were fixed and stained with 1 ml of 0.005% Crystal Violet for 1 hour. The photographs of the cells growing in the plate and the colonies developed in soft agar were taken, the number of colonies larger than 0.5 mm was scored by ImageJ V1.45 ( NIH , USA).

**Cell proliferation assays by CCK-8 or RTCA**

For cell proliferation assays by CCK-8, cells were plated in 96-well plates (3×10^3^ cells/ well) and cell proliferation was determined by Cell Counting Kit–8 (CCK-8) (Sigma-Aldrich, Shanghai, China) at indicated time points. For cell proliferation assays by RTCA, cells at a density of 3×10^3^ cells/ well were seeded to a E-plate and subjected to the xCELLigence RTCA-DP system for real-time cell growth analysis (RTCA) lasting for 24 h.

**Western blotting analysis**

Briefly, cells were washed with PBS twice and lysed in SDS buffer (62.5 mM Tris, 2% SDS, pH 6.8), boiled for 10 minutes, sonicated (4 seconds, repeated 6 times, 30% power) and centrifuged at 12000 g for 15 minutes. The supernatant fraction was removed, and the protein concentration was determined by NanoDrop 2000 Spectrophotometer (Thermo Scientific, USA). Equal amount of proteins (100~200 μg) were separated by electrophoresis on a 12% or 8% SDS-polyacrylamide gel and transferred onto PVDF membrane (Millipore, Bedford, MA). After blocking with 5% non-fat dry milk in TBST buffer for 1 hour, the membrane was incubated with primary antibodies against Twist1 (ab50887, ChIP Grade, abcam, 1:200), E-cadherin (#3195, Cell Signaling Biotechnology, 1:1000), N-cadherin (sc-8424, Santa Cruz Biotechnology, 1:200), Vimentin (V6630, Sigma, 1:1000), or β-actin (#4970, Cell Signaling Biotechnology, 1:5000), Dnmt3a (ab2850, ChIP Grade, abcam, 1:500), Dnmt3b (#2161, Cell Signaling Biotechnology, 1:1000), Dnmt1 (#5032, Cell Signaling Biotechnology, 1:1000) overnight at 4°C. After washing with TBST buffer, the membrane was incubated with secondary antibodies against rabbit immunoglobulin G or mouse immunoglobulin G. The signals were visualized by LASmini4000 (General Electric Company).

**ChIP assays**

Chromatin immunoprecipitation (ChIP) was performed using EZ-ChIP kit (#17-371, Millipore, Billerica, MA). The protocols are referred to the instruction manual. The dissociated DNA was detected by PCR with the following primer sequences:

ChIP-primer-1 for NF-κB binding site1:

ChIP-primer-1 F, GGCGCCTGTGAGTTTAGTTCTAGAA;

ChIP-primer-1 R, GCAGCTATGTCTTCACAGGAGGAAA;

ChIP-primer-2 for NF-κB binding site2/3:

ChIP-primer-2 F, AATTGATCAGTCACTTCCGCCTCCAC;

ChIP-primer-2 R, TTCTAGAACTAAACTCACAGGCGCC;

ChIP- control primer:

ChIP- control primer-1 F, ACCTAAACTGAACACGTGCAGAACAA;

ChIP- control primer-1 R, GTTCAGGTCAAAAGTTCGGTCTCATC;

ChIP primer for IκBα promoter :

Forward: GACGACCCCAATTCAAATCG;

Reverse: TCAGGCTCGGGGAATTTCC

ChIP primer for E-box1+E-box2:

Forward: CACTGCGTTACAACTGCCTACAGTA;

Reverse: CTTTCGGCAATTGTGCTGTTGTGTG;

ChIP primer for E-box3+E-box4:

Forward: CCAGCTGGTTCCCATGTATAAGTTGC;

Reverse：CTCGGGCTCTTCAAGTGTTTAGAGA;

ChIP primer for E-box5:

Forward: AATTGATCAGTCACTTCCGCCTCCAC;

Reverse: TTCTAGAACTAAACTCACAGGCGCC;

ChIP primer for Dnmt3a binding:

Forward:AATTGATCAGTCACTTCCGCCTCCAC;

Reverse: TATCTTTTCTAGAACTAAACTCACA

**DNA methylation analysis via** **methylation specific PCR (MSP)**

DNA from cell lines were puriﬁed using TIANamp Genomic DNA Kit (#DP304-02, Tiangen Biotech, Shanghai, China), and treated with sodium bisulﬁte using EZ DNA Methlyation-Direct^TM^ kit (#D5021, ZYMO RESEARCH, Irvine, CA, USA), and then analyzed by MSP. The primer sequences for MSP are listed as below:

MiR186-promotor-MSP-1-M-F: GGTTTTTCGAGGTTTTTAGTGTTAC;

MiR186-promotor-MSP-1-M-R: TAACCAAAAATTCAACTTATACGTT;

MiR186-promotor-MSP-1-U-F: TTTTTTGAGGTTTTTAGTGTTATGA;

MiR186-promotor-MSP-1-U-R: CTAACCAAAAATTCAACTTATACATT;

MiR186-promotor-MSP-2-M-F: GGTTTTTAAACGATATTTTATGTG;

MiR186-promotor-MSP-2-M-R: CAAAAATAAAATACTTCCGCTTCCT;

MiR186-promotor-MSP-2-U-F: TTTTTAAATGATATTTTATGTGTATG;

MiR186-promotor-MSP-2-U-R: CAAAAATAAAATACTTCCACTTCCT;

Twist1-MSP-1-U-F: TTTGGATGGGGTTGTTATTGT;

Twist1-MSP-1-U-R: CCTAACCCAAACAACCAACC;

Twist1-MSP-1-M-F: TTTCGGATGGGGTTGTTATC;

Twist1-MSP-1-M-R: AAACGACCTAACCCGAACG;

Twist1-MSP-2-M-F: AGTTCGTATTGAGAAGTTTATGAGC;

Twist1-MSP-2-M-R: CACAATATTAAAATAAAAACAACGTT;

Twist1-MSP-2-U-F: TTTGTATTGAGAAGTTTATGAGTGG;

Twist1-MSP-2-U-R: CACAATATTAAAATAAAAACAACATT.

**Sodium bisulfite sequencing**

DNA from cell lines were puriﬁed using TIANamp Genomic DNA Kit (#DP304-02, Tiangen Biotech, Shanghai, China), and treated with sodium bisulﬁte using EZ DNA Methlyation-Direct^TM^ kit (#D5021, ZYMO RESEARCH, Irvine, CA, USA). The treated DNA was ampliﬁed by PCR using the bisulﬁte-speciﬁc primers

BS-F:TTTATGTATATATAAAAATAGGTT BS-R:AAAAAACTAACCAAAAATTCAACTT

The puriﬁed PCR products were cloned into the pMD20-T vector (Takara), and ten clones were picked out and sequenced for each sample.

**MicroRNA *in situ* hybridization (ISH)**

Prostate cancer tissue array was purchased from ALenabio (Cat#PR8011a, Xi’an, China). The expression levels of miR186 in Clinical prostate cancer specimens was evaluated by *in situ* hybridization assay as described.[^9^](#_ENREF_9)

**REFERENCES**

1 Bae VL, Jackson-Cook CK, Brothman AR, Maygarden SJ, Ware JL (1994). Tumorigenicity of SV40 T antigen immortalized human prostate epithelial cells: association with decreased epidermal growth factor receptor (EGFR) expression. *International journal of cancer Journal international du cancer* **58:** 721-729.

2 Bae VL, Jackson-Cook CK, Maygarden SJ, Plymate SR, Chen J, Ware JL (1998). Metastatic sublines of an SV40 large T antigen immortalized human prostate epithelial cell line. *The Prostate* **34:** 275-282.

3 Chen C, Ridzon DA, Broomer AJ, Zhou Z, Lee DH, Nguyen JT *et al* (2005). Real-time quantification of microRNAs by stem-loop RT-PCR. *Nucleic Acids Res* **33:** e179.

4 Hayward SW, Dahiya R, Cunha GR, Bartek J, Deshpande N, Narayan P (1995). Establishment and characterization of an immortalized but non-transformed human prostate epithelial cell line: BPH-1. *In Vitro Cell Dev Biol Anim* **31:** 14-24.

5 Huang J, Yan J, Zhang J, Zhu S, Wang Y, Shi T *et al* (2012). SUMO1 modification of PTEN regulates tumorigenesis by controlling its association with the plasma membrane. *Nature communications* **3:** 911.

6 Liu X, Wang Y, Sun Q, Yan J, Huang J, Zhu S *et al* (2012). Identification of microRNA transcriptome involved in human natural killer cell activation. *Immunology letters* **143:** 208-217.

7 Liu X, Chen Q, Yan J, Wang Y, Zhu C, Chen C *et al* (2013). MiRNA-296-3p-ICAM-1 axis promotes metastasis of prostate cancer by possible enhancing survival of natural killer cell-resistant circulating tumour cells. *Cell death & disease* **4:** e928.

8 Qu Y, Chen Q, Lai X, Zhu C, Chen C, Zhao X *et al* (2014). SUMOylation of Grb2 enhances the ERK activity by increasing its binding with Sos1. *Molecular cancer* **13:** 95.

9 Tsai JH, Yang J (2013). Epithelial-mesenchymal plasticity in carcinoma metastasis. *Genes & development* **27:** 2192-2206.
