# Supplementary material for: Twist1/Dnmt3a and miR186 establish a regulatory circuit that controls inflammation-associated prostate cancer progression
Source: Oncogenesis. 2017 Apr 10;6(4):e315–. doi: 10.1038/oncsis.2017.16 (PMC5520493; doi:10.1038/oncsis.2017.16)
Supplement: Supplementary Figures [file oncsis201716x2.docx]

**SUPPLEMENTARY FIGURE LEGENDS**

Supplementary Figure 1**.** MiR186 regulates cell proliferation, migration and invasion. **(a, d)** RTCA growth assays for BPH1 and LT-BPH1 **(a)**, LT-BPH1-Vector and LT-BPH1-miR186 cells **(d)**. The kinetic migration curves were real-time recorded. Error bars indicate ± SD (related to Figure 1**d, j**). **(b, e)** RTCA migration assays for BPH1 and LT-BPH1 **(b)**, LT-BPH1-Vector and LT-BPH1-miR186 cells **(e)**. The kinetic migration curves were real-time recorded. Error bars indicate ± SD (related to Figure 1**e, k**). **(c, f)** RTCA migration assays for BPH1 and LT-BPH1 **(c)**, LT-BPH1-Vector and LT-BPH1-miR186 cells **(f)**. The kinetic migration curves were real-time recorded. Error bars indicate ± SD (related to Figure 1**f, l**).

Supplementary Figure 2**.** MiR186 is directly activated by NF-κB under inflammation signals. **(a-b)** The miR186 levels in BPH1 **(a)** or P69 **(b)** cells treated with TNFα (25ng/ml) for 0, 2, 4, 6, 12, 24 h were determined by real-time PCR. U6 was used as internal control. The experiments were performed at least three independent times, and error bars indicate±SEM, P-values of < 0.001 (***). **(c)** Luciferase activity analysis for the miR186 promoter activity in P69 cells treated with TNFα (25 ng/ml) for indicated time. The experiments were performed at least three independent times, and error bars indicate±SEM, P-values of<0.01 (**) and< 0.001(***). **(d-g)** Nuclear fractions extracted from BPH1 **(d)** and P69 **(f)** cells treated with LPS (1 μg/ml) for 0, 1, 2, 4, 6, 12, 24 h, and from BPH1 **(e)** and P69 **(g)** cells treated with TNFα (25 ng/ml) for 0, 0.5, 1, 2, 4, 6, 12 h, respectively. The levels of p-P65 (S536) and total P65 were analyzed by Western blotting. LaminB was used as nuclear loading control, and GAPDH was used as cytoplasmic control. **(h)** The luciferase activity analysis for the mutant miR186 promoter reporter containing double mutations (for NF-κB BS1&2) in P69 cells treated with LPS (1 μg/ml) for indicated time. The experiments were performed at least three independent times, and error bars indicate ± SEM. **(i)** Western blotting analysis for the p65 levels in BPH1 cells transfected with NC (control siRNA) or siP65-1/2 (related to Fig. 2**j**), and Real-time PCR analysis for the miR186 levels in P69-Ctrl or P69-siP65-2 cells treated with TNFα (25 ng/ml) for indicated different time (Right).

Supplementary Figure 3**.** The activation of miR186-Twist1 pathway in non-transformed prostate cells. **(a-f)** Five cell lines including BPH1 **(a, b)**, P69 **(c, d)** and P69-anti-miR186 **(e, f)** were respectively treated with 1 μg/ml LPS or 25 ng/ml TNFα for different time periods as indicated, and lysed for western blotting analysis of Twist1 and real-time PCR analysis of miR186. **(g)** The miR186 levels in PC3, P69 and BPH1 were determined by real-time PCR. U6 was used as internal control. The experiments were performed at least three independent times, and error bars indicate ± SEM, P-values of < 0.001 (***) **(h-i)** LT-BPH1 **(h)** nd PC3 **(i)** cells were respectively treated with 1 μg/ml LPS or 25 ng/ml TNFα for different time periods as indicated, corresponding western blots of Twist1 protein assessment was shown**.**

Supplementary Figure 4**.** The methylation of miR186 promoter is involved in the responsiveness to inflammatory signal. **(a)** A schematic diagram of CpG inlands in the ZRANB2/miR186 promoter region. The underlines indicate the fragments amplified by MSP primer1/2. **(b)** The methylation levels of CpG islands in the miR186 promoter in P69, M12, PC3 cells were measured by MSP analysis. ‘U’ represents unmethylated DNA products with non-methylation specific primers, and ‘M’ refers to methylated DNA products with methylation-speciﬁc primers. **(c-d)** LT-BPH1 cells treated with 5-aza-CdR were respectively treated with 1 μg/ml LPS **(c)** or 25 ng/ml TNFα **(d)** for different time periods as indicated, corresponding western blots of Twist1 protein assessment was shown**.** **(e-f)** RTCA migration **(e)** or invasion **(f)** for LT-BPH1 cells pre-treated with or without 5μM 5-aza-CdR for 24 h, and then subjected to a dynamic migration assay lasting for 28 h (continuing addition of 5-aza-CdR to the treated cells). The kinetic migration or invasion curves were real-time recorded (related to Fig. 3J-K). **(g)** A schematicdiagram of the Twist1 promoter region. The underlines indicate the fragments (CpG inland regions) amplified by MSP primer1/2. TSS: transcription start site. The methylation levels of the Twist1 promoter in BPH1, LT-BPH1, LT-BPH1 pretreated with DMSO or 5-aza-CdR for 48 h, P69, M12 and PC3 cells were determined by MSP analysis with primer1 and primer2. ‘U’ represents unmethylated DNA products with non-methylation speciﬁc primers, and ‘M’ refers to methylated DNA products with methylation-speciﬁc primers.

Supplementary Figure 5**.** Twist1 represses miR186 in a negative feedback loop throught directly interacting with and recruiting Dnmt3a to the miR186 promoter. **(a)** Luciferase activity analysis for miR186 promotor activity in 293T cells transfected with increasing amounts of Twist1 expression. The experiments were performed at least three independent times, and error bars indicate ± SEM, P-values of < 0.05 (*), < 0.01 (**) and < 0.001 (***). **(b)** Lysates from LT-BPH1 cells were used for co-IP with the monoclonal antibody anti-Dnmt3a or anti-IgG, and followed by immunoblotting with antibodies against Dnmt3a and Twist1. **(c)** Lysates from 293T cells transfected with Myc-Dnmt3a were pull-down with purified GST and GST-Twist1, and immunoblotted with the antibodies anti-Myc and anti-GST. **(d)** Lysates from 293T transfected with Myc-Dnmt3a were pull-down with purified GST, GST-Twist1(full-length), GST-Twist1(1-107) or GST-Twist1(108-203) on glutathione sepharose beads, and detected with the antibody anti-Myc (Top panel); A schematic representation of Twist1 truncated according to different functional domains (Bottom panel). **(e)** Lysates from 293T transfected with full-length Myc-Dnmt3a or truncated Myc-Dnmt3a were pull-down with purified GST or GST-Twist1(full-length) on glutathione sepharose beads, and detected with the antibody anti-Myc ( Top panel ); A schematic representation of Dnmt3a truncated according to different functional domains (Bottom panel).

Supplementary Figure 6**.** Twist1 recruiting Dnmt3a facilitates the site-specific CpG methylation of the miR186 promoter. **(a)** Sequence mapping of NF-κB binding sites (BSs, in red or yellow), Twist1 binding E-boxes (in blue), and CpG inlands at the miR186 promoter (-289 to +290) region. TSS represents miR186 transcriptional start site, and CpG is black highlighted and labeled by numbers. **(b)** ChIP analysis for Dnmt3a occupancy at miR186 promoter in BPH1 cells expressing control vector, Twist1 and shRNA specific for Twist1. **(c)** Western-blot analysis for Dnmt3a protein levels by the first ChIP (1 st ChIP), and the Twist1 protein levels by reChIP (2 nd ChIP).

Supplementary Figure 7**.** A double-negative feedback regulatory circuit controls inflammation-associated prostate cancer progression. In short, under moderate inflammatory stimulation, the NF-κB signaling pathway activation accelerated miR186 transcription through directly binding to the NF-κB BS2 in the miR186 promoter in non-transformed cells. NF-κB also up-regulated Twist1 expression by directly binding its promoter, but a large number of Twist1 transcripts was degradated or translationally inhibited by miR186. So, in this situation, Twist1 protein remained a low level and cells appeared to be self-healing in inhibition of cell malignant transformation. On the contrary, when inflammation persisted, Twist1 downregulated miR186 in a novel negative feedback loop through directly binding to the E-box and simultaneously recruiting Dnmt3a to facilitate the site-specific CpG methylation of the miR186 promoter, thereby these blocked the transcriptional activity of NF-κB/P65 and the responsiveness of miR186 to inflammatory signals. At the same time, the activated NF-κB/p65 greatly promoted Twist1 transcription, leading to the accumulation of intracellular Twist1 protein into an irreversible vicious cycle, in turn aggregately repressing miR186 expression and thereby perpetuating the cell transformed state.
