# Supplementary figures and images for: Twist1/Dnmt3a and miR186 establish a regulatory circuit that controls inflammation-associated prostate cancer progression
Source: Oncogenesis. 2017 Apr 10;6(4):e315–. doi: 10.1038/oncsis.2017.16 (PMC5520493; doi:10.1038/oncsis.2017.16)

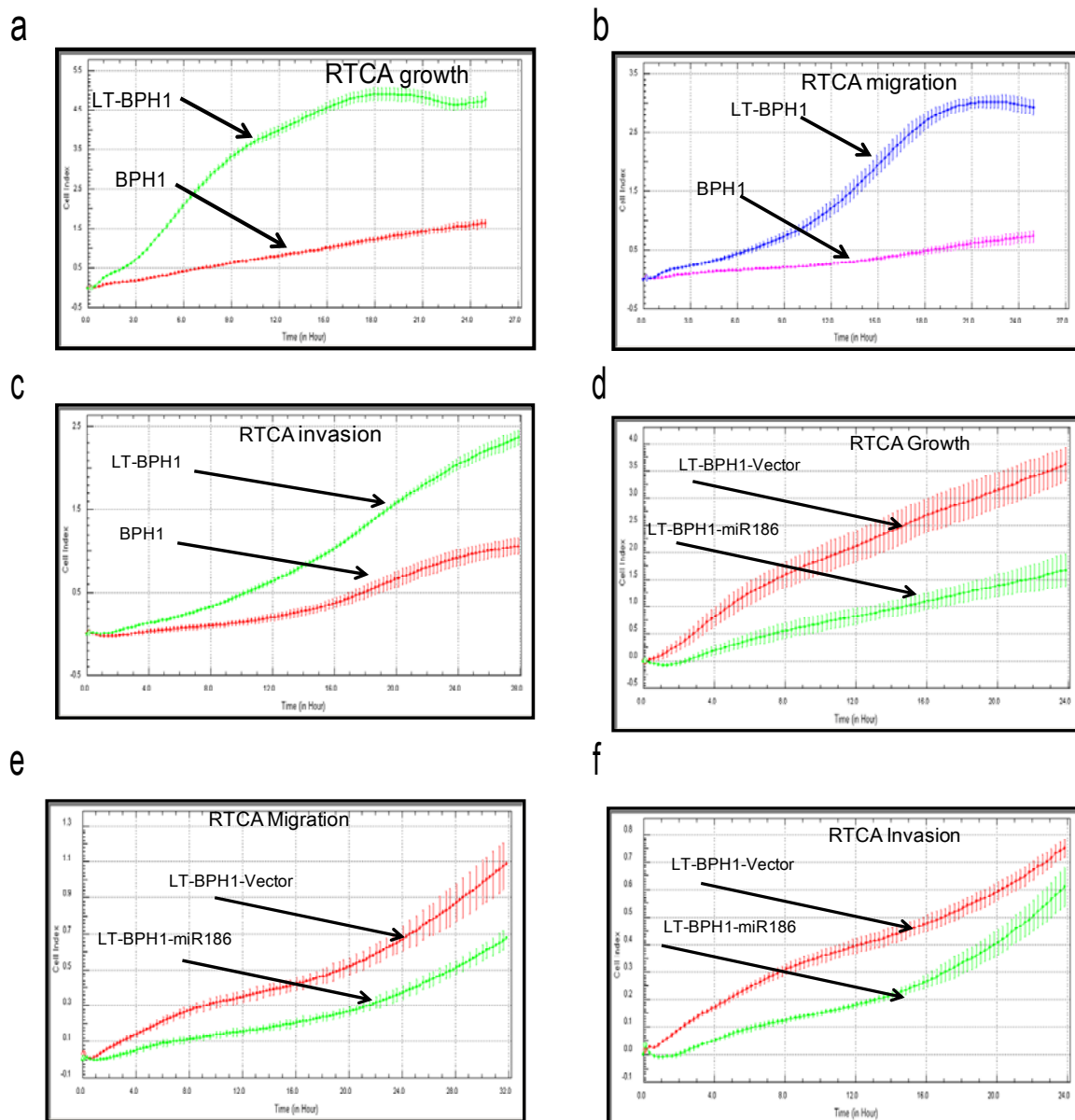

**Figure S1**

Supplement: Supplementary Figure 1 [file oncsis201716x3.pdf]

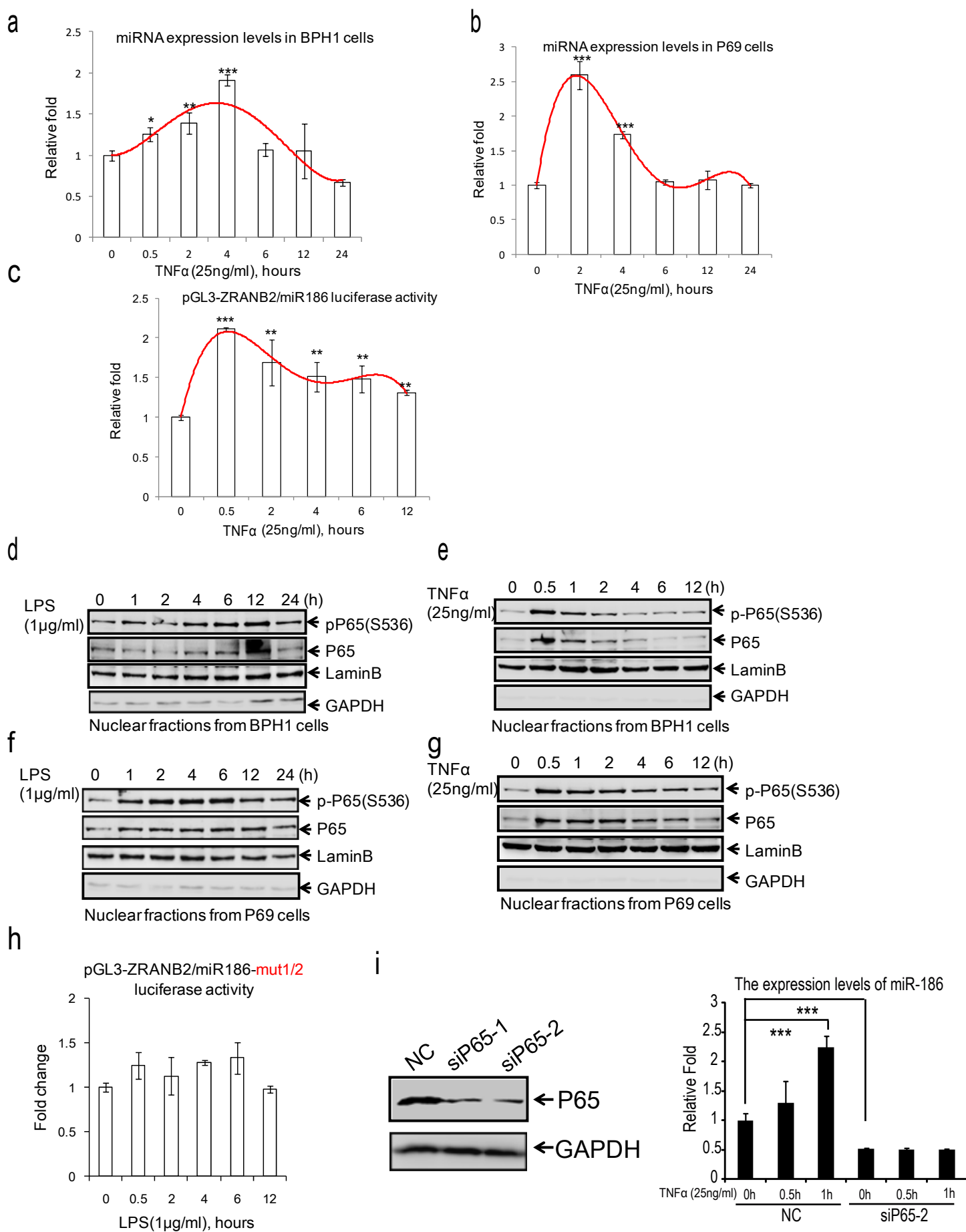

**Figure S2**

Supplement: Supplementary Figure 2 [file oncsis201716x4.pdf]

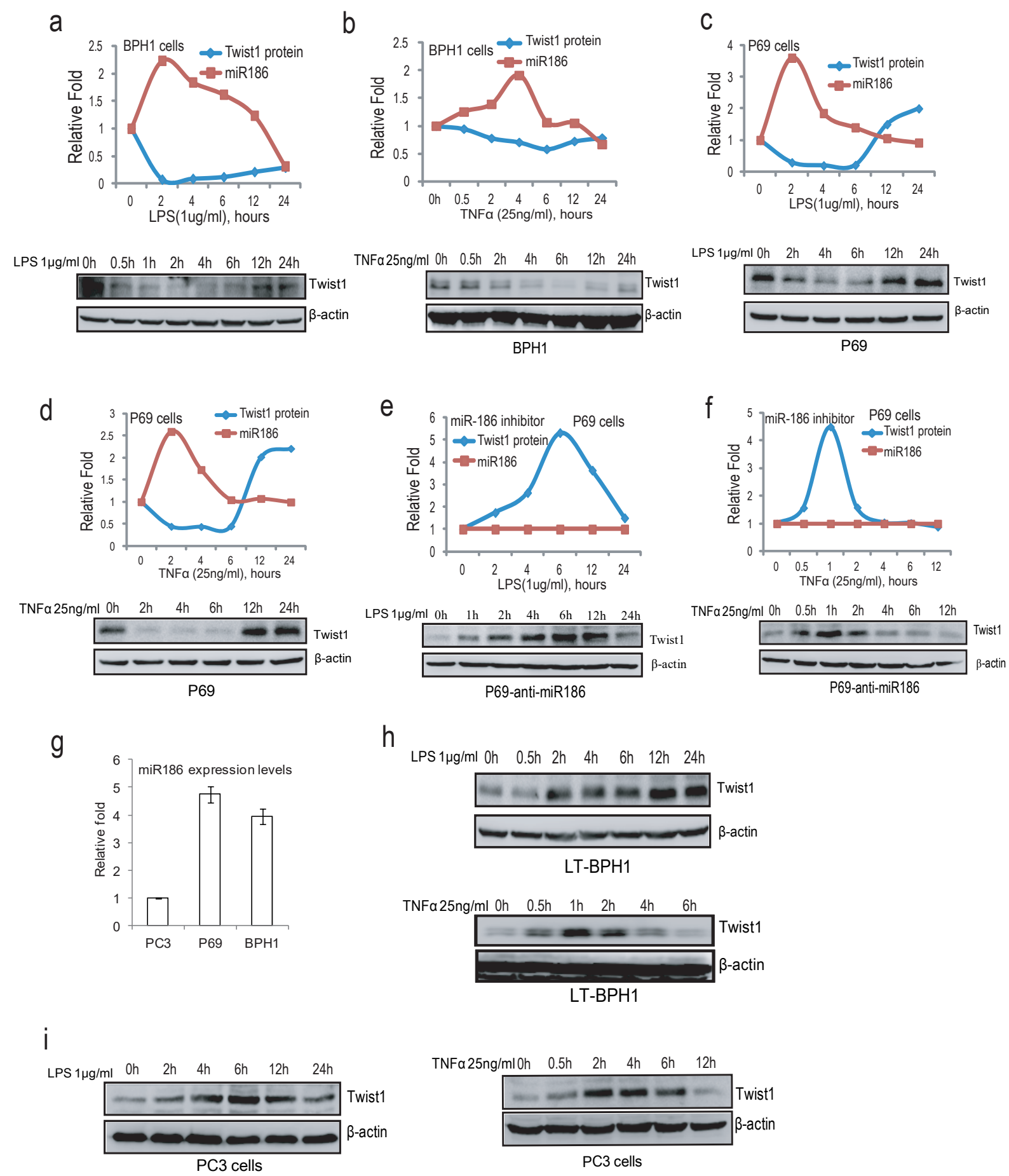

**Figure S3**

Supplement: Supplementary Figure 3 [file oncsis201716x5.pdf]

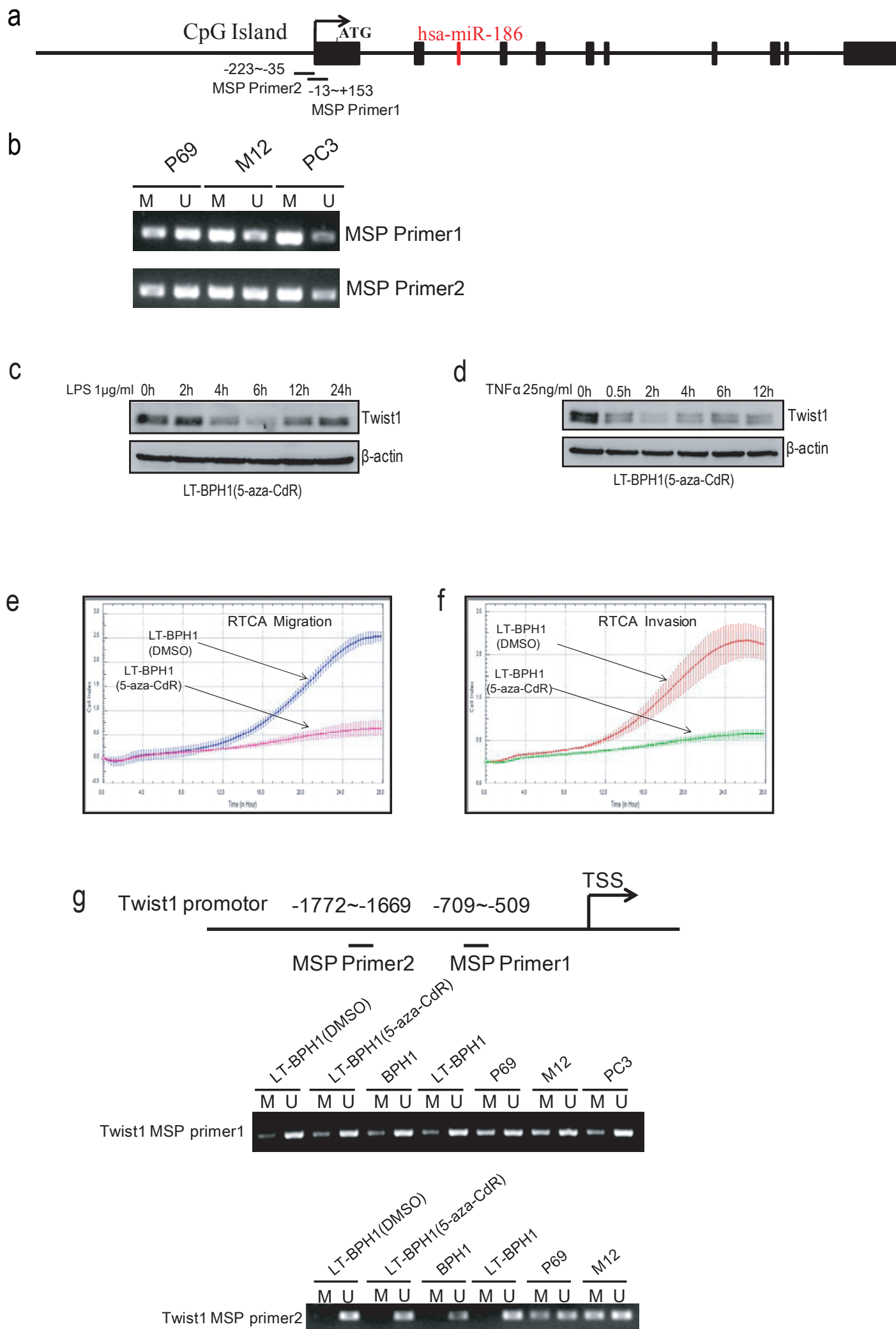

**Figure S4**

Supplement: Supplementary Figure 4 [file oncsis201716x6.pdf]

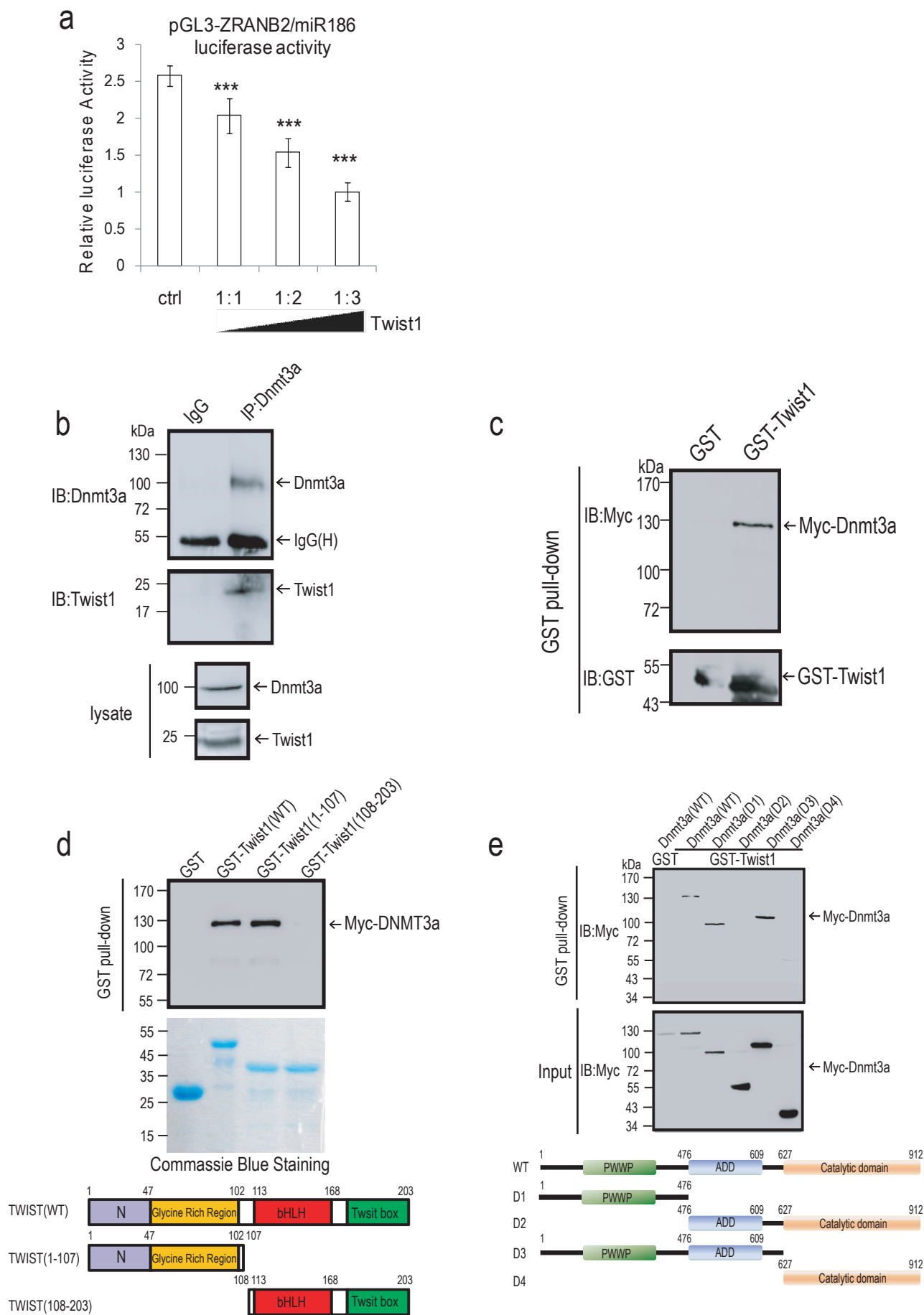

## Figure S5

Supplement: Supplementary Figure 5 [file oncsis201716x7.pdf]

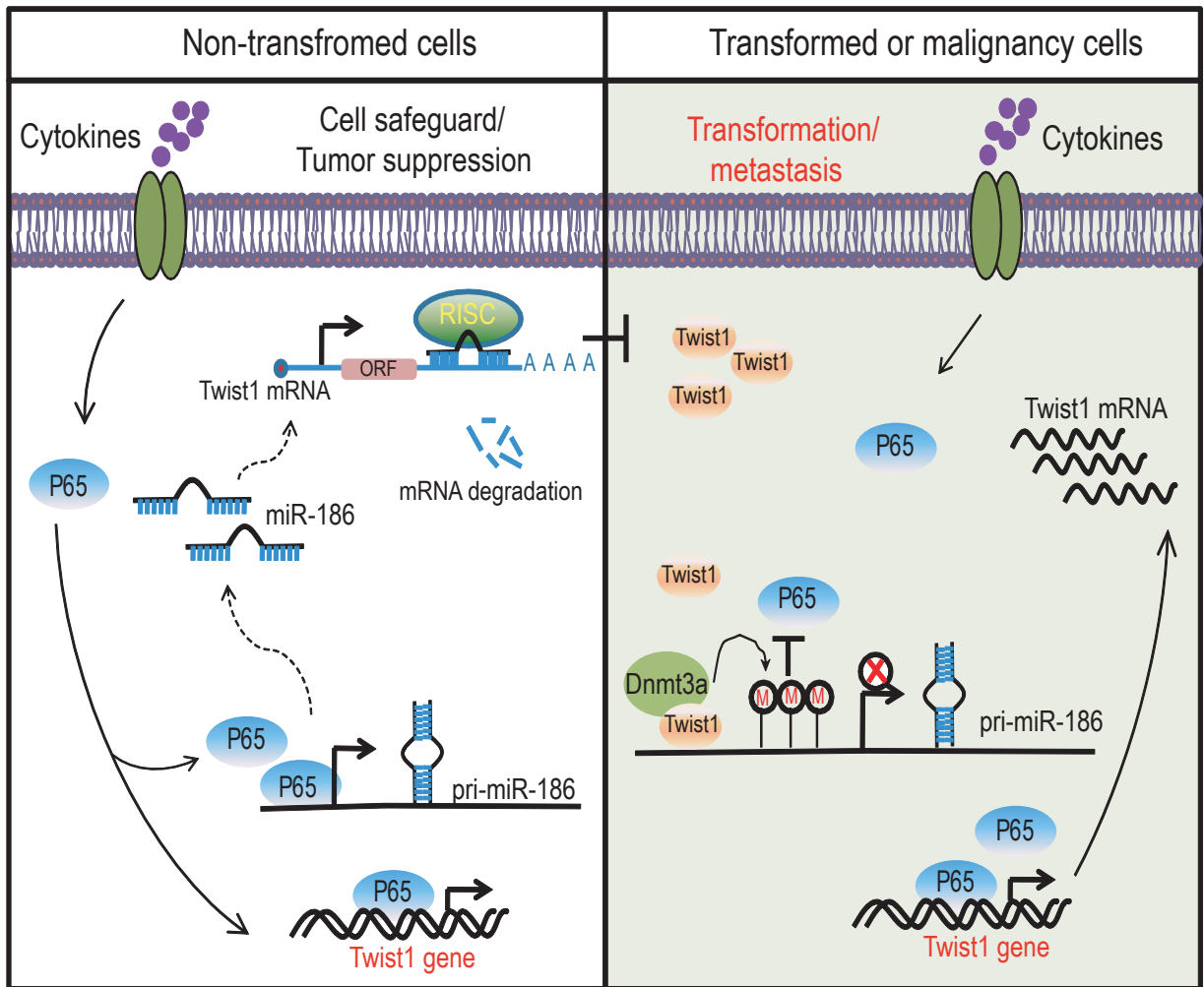

**Figure S7**

Supplement: Supplementary Figure 7 [file oncsis201716x9.pdf]
