# Supplementary material for: Twist1/Dnmt3a and miR186 establish a regulatory circuit that controls inflammation-associated prostate cancer progression
Source: Oncogenesis. 2017 Apr 10;6(4):e315–. doi: 10.1038/oncsis.2017.16 (PMC5520493; doi:10.1038/oncsis.2017.16)
Supplement: Supplementary Figure 6 [file oncsis201716x8.pdf]

a

Twist1 BS /E3

CCAGCTGGTTCCCATGTATAAGTTGCTTACTTATCTGTTACACCTATTTATGTATA

TATAAAAATAGGTTTCCAAACG[1]ACATCCCATGTGTATGAAATAACAGAATTTTTAT

Twist1 BS /E4

GCCTTATTAAAATAATTTCTCTAAACACTTGAAGAGCCCG[2]AGCCG[3]CCTCTTACC

CTAAACG[4]AACCCCACG[5]CCCCTGAATTGATCAGTCACTTCCG[6]CCTCCACCTTTC

NF-κB BS3 Twist1 BS /E5

CAGCTCCACCTAAGGAAGCG[7]GAAGTACTTTACTCTTGGGTCCTCCCACCTGTAG

NF-κB BS2 TSS

GGGGCTCTCCG[8]AGGCTTCTAGTGCCACG[9]AAGGCCTCCTGCCTGGGTTGTCCT

TATGGCG[10]CCTGTGAGTTTAGTTCTAGAAAAGATATTTGTATTTTAACTTTTCCAT

CTAACAAACAGTAGCACAGGCATCTTAGTCTGGACAACAACG[11]TACAAGCTGAAT

TCCTGGCTAGTTCCCTCATTATCAAACCCTGACACAGTAGTAGGCG[12]CG[13]AGTC

NF-κB BS1

AGGCAGTGTGGGGACAGCCCCG[14]AGTTGCCG[15]AAAGTCCCCG[16]GCCCG[17]TTTT

CCTCCTGTGAAGACATAGCTGCG[18]GGTGGCTGTGCTGGTGGCG[19]TTCAAG

b

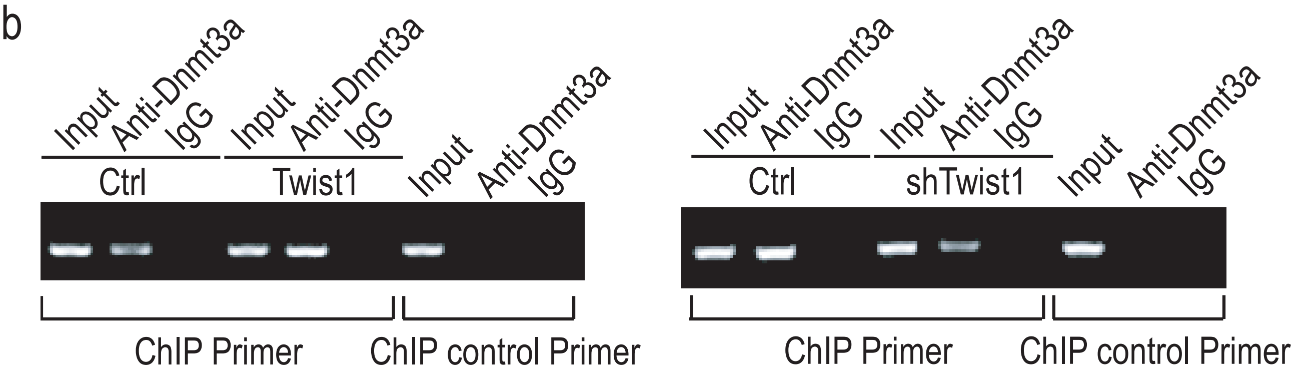

c

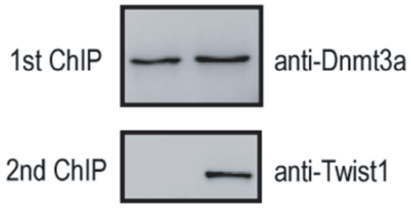

Figure S6
